# Unprecedented frequency of mitochondrial introns in colonial bilaterians

**DOI:** 10.1038/s41598-022-14477-3

**Published:** 2022-06-28

**Authors:** Helen Louise Jenkins, Rachael Graham, Joanne Sara Porter, Leandro Manzoni Vieira, Ana Carolina Sousa de Almeida, Andrea Hall, Aaron O’Dea, Simon Edward Coppard, Andrea Waeschenbach

**Affiliations:** 1 Department of Life Sciences, Natural History Museum, Cromwell Road, London, SW7 5BD UK; 2 Marine Biological Association, The Laboratory, Citadel Hill, Plymouth, PL1 2PB Devon UK; 3 International Centre Island Technology, Heriot Watt University, Orkney Campus, Robert Rendall Building, Franklin Road, Stromness, Orkney, KW16 3AW Scotland UK; 4 Laboratório de Estudos de Bryozoa—LAEBry, Departamento de Zoologia, Centro de Biociências, Universidade Federal de Pernambuco, Recife, PE 50670-901 Brazil; 5 Museu de História Natural, Setor da Zoologia, Universidade Federal da Bahia, Salvador, BA 40170-115 Brazil; 6 Core Research Labs, Natural History Museum, Cromwell Road, London, SW7 5BD UK; 7 Smithsonian Tropical Research Institute, Balboa, Ancon Republic of Panama; 8 Bader International Study Centre, Queen’s University (Canada), Herstmonceux Castle, Hailsham, BN27 1RN East Sussex UK

**Keywords:** Mitochondrial genome, Molecular evolution

## Abstract

Animal mitogenomes are typically devoid of introns. Here, we report the largest number of mitochondrial introns ever recorded from bilaterian animals. Mitochondrial introns were identified for the first time from the phylum Bryozoa. They were found in four species from three families (Order Cheilostomatida). A total of eight introns were found in the complete mitogenome of *Exechonella vieirai*, and five, 17 and 18 introns were found in the partial mitogenomes of *Parantropora penelope*, *Discoporella cookae* and *Cupuladria biporosa*, respectively. Intron-encoded protein domains reverse transcriptase and intron maturase (RVT-IM) were identified in all species. Introns in *E. vieirai* and *P. penelope* had conserved Group II intron ribozyme domains V and VI. Conserved domains were lacking from introns in *D. cookae* and *C. biporosa*, preventing their further categorization. Putative origins of metazoan introns were explored in a phylogenetic context, using an up-to-date alignment of mitochondrial RVT-IM domains. Results confirmed previous findings of multiple origins of annelid, placozoan and sponge RVT-IM domains and provided evidence for common intron donor sources across metazoan phyla. Our results corroborate growing evidence that some metazoans with regenerative abilities (i.e. placozoans, sponges, annelids and bryozoans) are susceptible to intron integration, most likely via horizontal gene transfer.

## Introduction

A crucial step in early eukaryote evolution was the origin of mitochondria, which arose by incorporating α Proteobacteria endosymbionts as cellular organelles^1^. Since this origin, the various evolutionary trajectories of eukaryotes have produced mitochondrial (mt) genomes (mitogenomes) that vary substantially in their sizes, gene content, genetic code, organization and physical shape^2^. Plant, fungal and other non-metazoan eukaryote mitogenomes range in size from 12 to 236 Kb, 66–11.3 Mb and 34–113 Kb, respectively and harbour a large proportion of non-coding DNA, including introns. In contrast, bilaterian mitogenomes are typically circular, streamlined molecules of approximately 16 kb (range: 11–50 Kb), which are typically devoid of introns^3^. The lack of introns in bilaterian mitogenomes has been attributed to the relatively high mutation rate of animal mtDNA, which can be ~ 9 to 25 times faster than that of corresponding nuclear DNA^4^. Introns, like all non-coding DNA, provide a substrate for potentially harmful mutations in the presence of high mutation rates, and are therefore hypothesised to have been purged from most animal mitogenomes^4,5^.

In addition to spliceosomal introns, which occur in the nuclear genomes of eukaryotes and require ribonucleoprotein complexes called spliceosomes to facilitate their excision from precursor mRNA, there are two other types of introns. Group I introns occur in bacterial, archaeal, viral and organellar genomes as well as some eukaryote nuclear genomes^6–9^. Group II introns occur in bacterial, archaeal and organellar genomes (particularly of plants, fungi, algae and protists), but are not found in nuclear genomes^10,11^. Both types are self-splicing, mobile ribozymes, but they are distinguished by their splicing mechanisms. Group I introns use external guanosine nucleotides as cofactors, whereas Group II introns use a mechanism more akin to that found in spliceosomal introns^8^ and are believed to be precursors of eukaryote spliceosomal introns, retroelements and spliceosome components^11,12^. Both types frequently harbour intron-encoded proteins (IEPs) that facilitate intron mobility; Group I introns typically encode homing endonucleases, whereas Group II IEPs include reverse transcriptase (RVT) and intron maturase (IM) domains^12^. In terms of their secondary structures, Group II introns form six domains (DI—DVI) that radiate out from a central loop. Based on the intricacies of their secondary structures, five Group II introns have been identified (IIA1, IIA2, IIB1, IIB2, IIC)^13,14^. Conversely, Group I introns typically form secondary structures of P1–P10 stems^15^.

Although typically not found in metazoans, both intron types have been recorded in the mitogenomes of non-bilaterian phyla Porifera^16–22^ and Placozoa^23–26^, whereas Cnidaria are only known to harbour Group I introns^27–29^. Conversely, in bilaterians, mitogenome introns are apparently very rare, but Group II introns have been recognised in the cox1 gene of annelids *Nephtys* (Phyllodocida)^30^, *Glycera fallax*, *G. unicornis* (Phyllodocida)^31^, *Decemunciger* sp. (Terebellida)^32^ and the myzostomid *Endomyzostoma* sp.^33^. Furthermore, high-throughput DNA sequencing has revealed additional introns of unknown type(s) in other protein-coding genes (PCGs) of *Decemunciger* sp. (nad1 and nad4^32^) and the mollusc *Cucullaea labiata* (Arcoida) (cox1^34,35^).

Here, we reveal the widespread occurrence of introns in the mitogenomes of taxa of the lophotrochozoan phylum Bryozoa. Bryozoans, whose fossil record goes back as far as the Cambrian^36^, are modular invertebrates, found worldwide in aquatic habitats, including freshwater bodies, shallow coastal waters, and the deep sea^37,38^. Almost exclusively colonial, they form encrustations on a variety of substrates, construct erect, three-dimensional structures or produce free living disks^39,40^. There are three classes of bryozoans. The least diverse of these is the exclusively freshwater class Phylactolaemata with ~ 86 Recent species^41^. The Phylactolaemata forms the sister group to all remaining bryozoans^42^. The class Stenolaemata, whose only surviving order is the exclusively marine Cyclostomatida, constitutes ~ 543 Recent species^41^ and they form the sister group to the class Gymnolaemata^42^. Although predominantly marine, the Gymnolaemata also includes a few brackish and freshwater species. It is the most speciose class with their ~ 5240 Recent species, of which ~ 319 belong to the order Ctenostomatida and ~ 4921 belong to the order Cheilostomatida^41^; the Cheilostomatida nest within a paraphyletic Ctenostomatida^42^. Apart from phylactolaemates, which can disperse via asexually produced propagules called statoblasts, and some gymnolaemates which can form resting structures called hibernacula^43^, bryozoan colonies are typically established by sexually produced larvae, which settle and metamorphose into the founding module (zooid) of the colony, the ancestrula^37^. The ancestrula then buds the adjacent zooids, which in turn continue the colony growth via asexual budding of further zooids^44^. Reparative growth of damaged colony parts frequently occurs^45^, and some species propagate largely via colony fragmentation and subsequent regeneration^46^.

**Figure 1:**
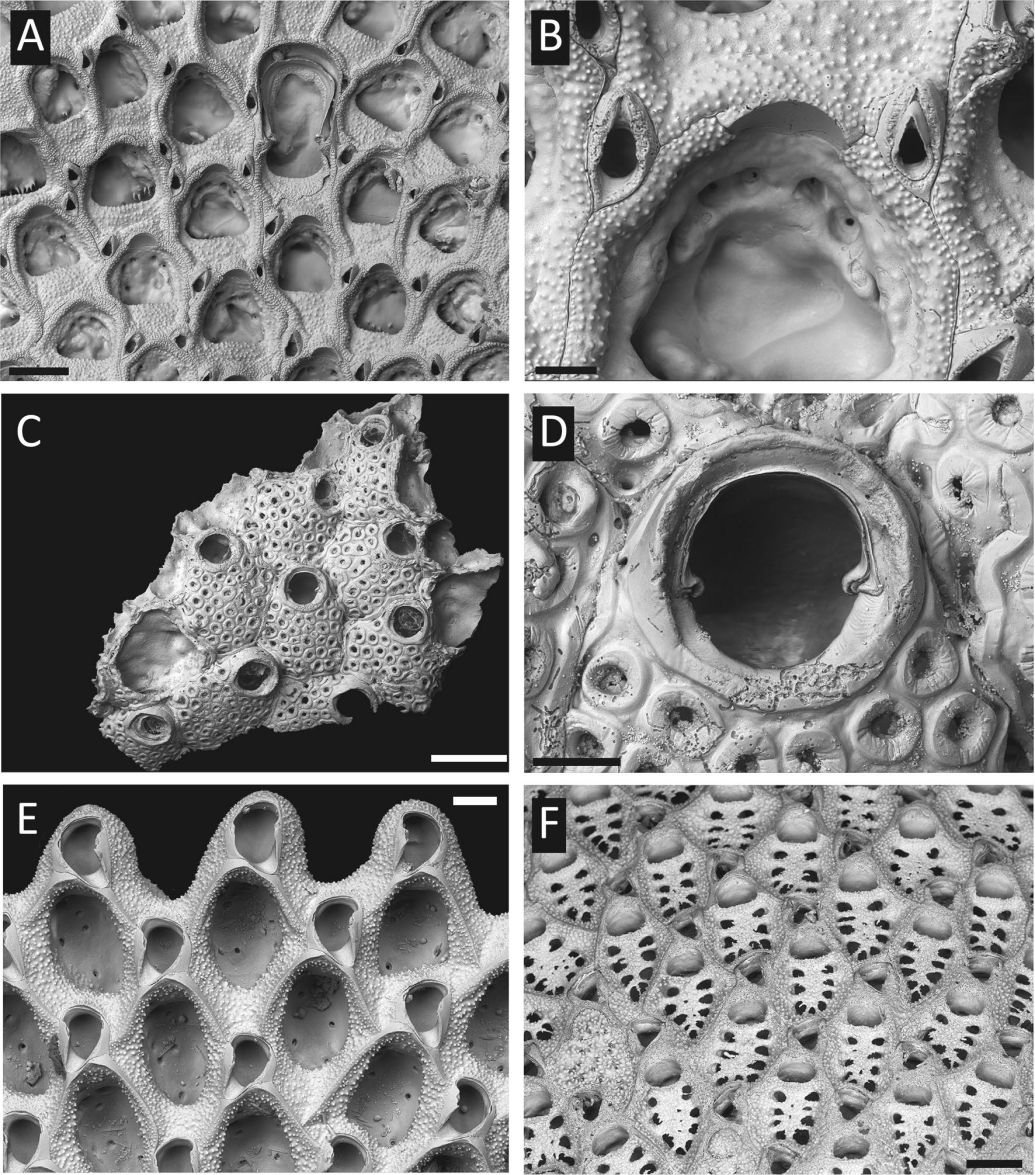
Scanning electron microscope images of morphological voucher specimens. *Parantropora penelope* (NHMUK 2021.2.9.1.) (**A,B**), *Exechonella vieirai* (NHMUK 2018.5.17.81) (**C,D**), *Cupuladria biporosa* (NHMUK 2018.5.17.101) (**E**), *Discoporella cookae* (NHMUK 2021.2.9.2*.*) (**F**). Scale bars = 200 µm (**A**), 50 µm (**B**), 500 µm (**C**), 100 µm (**D,E**), 200 µm (**F**).

At present, there are eight verified published bryozoan mitogenomes available from NCBI (https://www.ncbi.nlm.nih.gov/), none of which contain introns (see GenBank accessions NC_008192, NC010197, NC_011820, NC_015646, NC_016722, NC_018344, NC_018355, NC_038192). As part of a wider study sequencing hundreds of mitogenomes from species of the bryozoan order Cheilostomatida, genome-skimming has revealed the occurrence of introns in multiple PCGs in single mitogenomes, multiple introns within single genes, as well as in intergenic regions. Here, we document introns discovered in the mitogenomes of four cheilostome bryozoans: two encrusting species – *Parantropora penelope* (AW2102; Calloporoidea, Antroporidae) and *Exechonella vieirai* (AW1260; Arachnopusioidea, Exechonellidae) and two free-living species from the family Cupuladriidae (Calloporoidea), *Cupuladria biporosa* (AW817) and *Discoporella cookae* (AW3739) (Fig. 1). Given the high frequency of introns recorded, we verify their presence using polymerase chain reactions (PCRs), in order to rule out mis-assemblies of Illumina reads being the cause of the fragmented open reading frames (ORFs). Furthermore, we analyse the PCGs in a phylogenetic context together with published RVT-IM domains to identify possible intron donor organisms. This is the largest number of mt introns reported from a bilaterian phylum, to date.

## Results and discussion

### Mitogenome information

**Figure 2:**
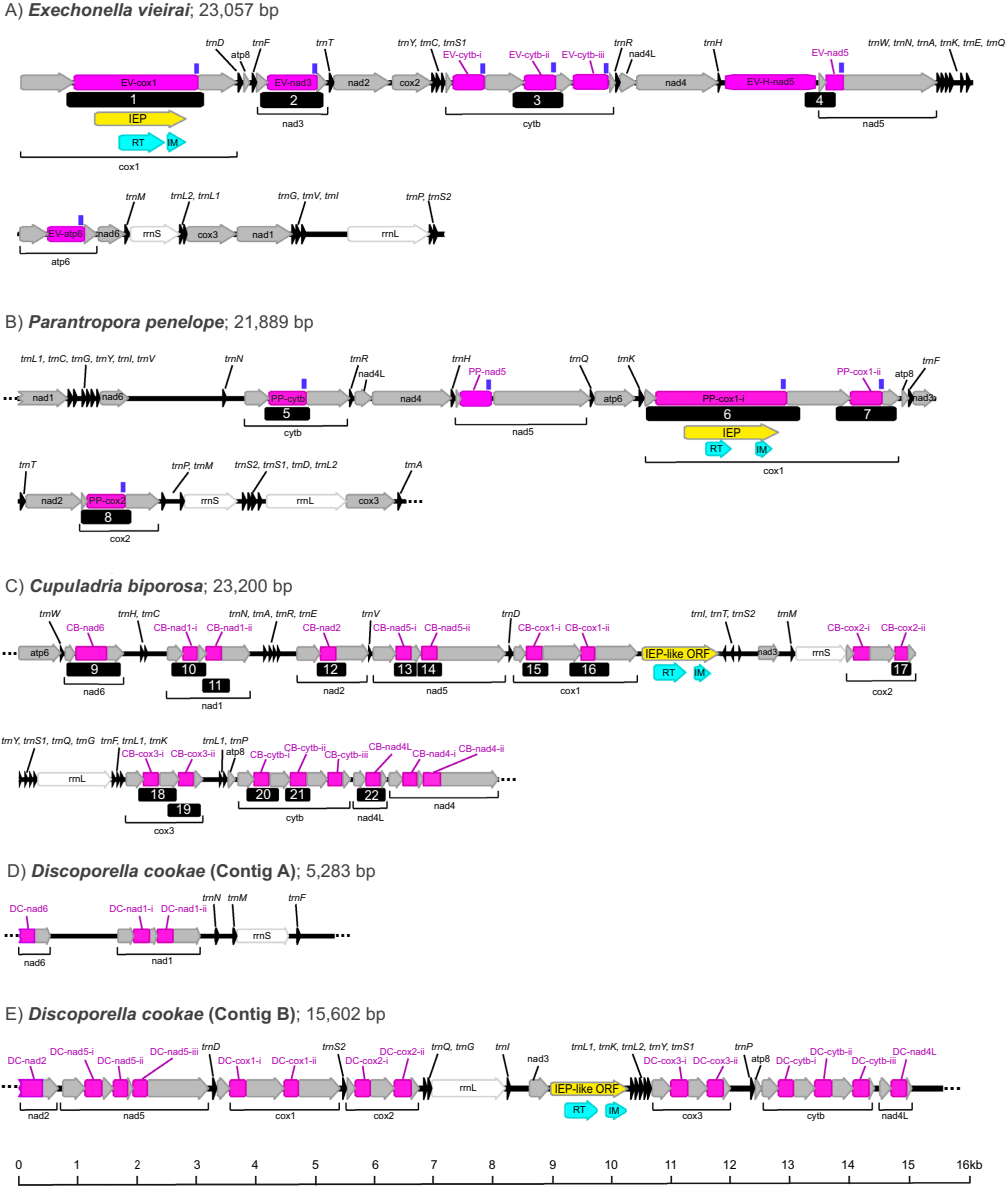
Mitogenome maps showing gene order and PCR product locations (black boxes 1–22). Linearised complete mitogenome of *Exechonella vieirai* (**A**). Partial mitogenome of *Parantropora penelope* (**B**). Non-circularised mitogenome of *Cupuladria biporosa* (**C**). Contig A of *Discoporella cookae* (**D**). Contig B of *Discoporella cookae* (**E**). Introns are labelled as pink boxes. Conserved Group II ribozyme domains V and VI, as found by Rfam, are indicated by vertical blue bars. Capital prefixes in intron labels represent genus/species initials. Whenever multiple introns were found per gene, introns were labelled with suffixes i-iii. Open reading frames (ORFs) of intron-encoded proteins (IEPs) are shown as yellow arrows. In the cases of *C. biporosa* and *D. cookae*, these ORFs are labelled as ‘IEP-like ORFs’ because they do not nest within introns. ORFs of reverse transcriptase (RT) and intron maturase (IM) are shown as turquoise arrows.

Numbers of raw Illumina reads per species, contig sizes and coverage, and GenBank accession numbers are given in Table 1. Only the mitogenome of *E. vieirai* could be circularised. In this mitogenome, genes nad1 and nad4 were terminated by abbreviated stop codon U (Supplementary Table S1); stop codon UAA is presumably completed by polyadenylation of the cleaved polycistronic transcript mRNA^47^. The mitogenome of *C. biporosa*, although complete with regards to gene contents, could not be circularised. Both these mitogenomes had the full complement of 13 PCGs, two rRNA genes and 22 tRNA genes. The mitogenomes of *P. penelope* and *D. cookae* were incomplete. Genes missing in the *P. penelope* contig were tRNAs trnW and trnE. Furthermore, we infer that gene nad4L starts on the non-standard initiation codon GUU, which encodes for valine (Supplementary Table S1). Codon GUG, also encoding for valine, has been shown to be a functional initiation codon for atp6 in some humans^48^ and has therefore been accepted as alternative initiation codon. However, no evidence for the functionality of GUU as initiation codon has yet been shown. The mitogenome data for *D. cookae* was in two separate contigs: Contig A (size: 5,283 bp) and Contig B (size: 15,602 bp). Missing genes were: PCGs atp6 and nad4 and tRNAs trnA, trnC, trnE, trnH, trnR, trnT, trnV and trnW; genes nad2 and nad6 were incomplete at their 5’ ends. For diagrammatic representation of gene order of mt fragments, see Fig. 2. For more detailed mitogenome information (gene boundaries/lengths, initiation/stop codons, GC content), see Supplementary Table S1.

**Table 1:** Number of raw Illumina reads, mitogenome contig sizes, coverage depth and GenBank accession numbers for *Exechonella vieirai*, *Parantropora penelope, Cupuladria biporosa* and *Discoporella cookae*.

	No. paired-end reads (pre- and post-trimming)	Mitogenome contig size (bp)	Circularised (Y/N)	Mean coverage	GenBank accession
*Exechonella vieirai*	3,979,105 3,656,084	23,057	Y	51.6×	MW592986
*Parantropora penelope*	4,832,737 4,419,735	21,889	N	16.2×	MW592988
*Cupuladria biporosa*	8,038,861 7,784,830	23,200	N	132×	MW592987
*Discoporella cookae* Contig A	13,944,941 13,520,261	5,283	N	18.4×	MW592990
*Discoporella cookae* Contig B		15,602		18.2×	MW592989

### Intron verification and characterization

A large number of introns were found within several PCGs, sometimes multiple introns per gene, across multiple species of cheilostome bryozoans (Fig. 2, Supplementary Table S2). We eliminate the possibilities of them being assembly artefacts by verifying 22 of them with PCRs and Sanger sequencing. Each of the introns examined with PCRs were confirmed to be bona fide. PCR products were of expected sizes (Supplementary Fig. S1) and flanking Sanger reads matched the existing assemblies.

The mitogenome of *E. vieirai* harboured single introns in three genes (EV-cox1, EV-nad3, EV-atp6; intron name prefixes indicate genus/species initials; Fig. 2, Supplementary Table S2). Furthermore, three introns were found in cytb (EV-cytb-i-iii) and there was one intron-like region situated between tRNA histidine and nad5 (EV-H-nad5) and one intron in nad5 (EV-nad5). The concatenation of EV-H-nad5 + EV-nad5 aligned well with other introns. Thus, it seems that this intron is interrupted by a short nad5 exon of 114 bp (Fig. 2; Supplementary Table S1). To eliminate the possibility of this being an assembly artefact, we confirmed the presence of this exon by PCR with primers annealing in flanking intron regions (Fig. 2). The PCR product was of the expected size of 607 bp (Supplementary Fig. S1) and Sanger reads matched the existing assembly. Thus, this is a bona fide result. To confirm that the short 114 bp exon is indeed part of nad5, we examined this region in the context of a wider alignment of published and unpublished bryozoan nad5 sequences. Although the 5’ end of nad5 is rather variable, we conclude that the 114 bp exon does fit into the alignment lengthwise and sequence-wise (see Supplementary Fig. S2). This result, however, raises the question on how intron-splicing and translation of the exon can be performed effectively seeing that the intron may not be able to fold into a functional secondary structure. This issue ought to be revisited in future work. The mitogenome of *P. penelope* had single introns in cox2 (PP-cox2), cytb (PP-cytb) and nad5 (PP-nad5) and two introns in cox1 (PP-cox1-i-ii) (Fig. 2; Supplementary Table S2). Furthermore, both *E. vieirai* and *P. penelope* had IEPs with RVT and IM domains in cox1 introns, EV-cox1 and PP-cox1-i, respectively (Fig. 2, Supplementary Tables S1 and S2; see Supplementary Fig. S3 for Pfam results).

The largest number of introns, however, was found in the two members of the family Cupuladriidae: *C. biporosa* and *D. cookae* Although there are substantial similarities in the intron distribution across these two species (two introns each in cox1, cox2, cox3, nad1; single introns in nad2, nad4L and nad6 [but note that the *D. cookae* fragments start on incomplete nad2 and nad6 sequences, so there may be more undiscovered introns]), *D. cookae* harboured three introns in nad5, whereas *C. biporosa* harboured only two introns in this gene. A further two introns were found in nad4 in *C. biporosa*, but due the partial nature of the *D. cookae* mitogenome, nad4 was missing from its contigs, thus, no inferences can be made about shared introns for this gene. Furthermore, both species have one intergenic region that harbours an open reading frame containing RVT and IM domains (for Pfam results, see Supplementary Fig. S3). In *C. biporosa* this is located between cox1 and tRNA isoleucine and in *D. cookae* it is found between nad3 and tRNA leucine 1 (Fig. 2; Supplementary Tables S1 and S2; see Supplementary Fig. S3 for Pfam results).

**Figure 3:**
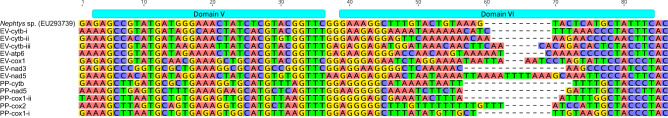
Group II ORF-less ribozyme domains V and VI alignments of *Exechonella vieirai* and *Parantropora penelope* with reference sequence *Nephtys* sp. (domain annotation from Fig. 1 in^30^).

Group II introns typically exhibit a secondary structure composed of six domains^13^. An alignment to reference sequence *Nephtys* sp. in which all six domains were annotated according to Vallès et al*.*^30^, revealed that all introns in *E. vieirai* and *P. penelope* had Group II intron ribozyme domains V and VI, as determined by Rfam searches (Supplementary Table S3), all of which aligned well with reference sequence *Nephtys* sp. (Fig. 3). None of the cupuladriid introns harboured conserved Group II ribozyme domains.

All but one intron of *E. vieirai* and *P. penelope*, started and ended on conserved splice motifs GUGYG and YAY^12,13^. PP-cox1-ii intron deviated from this pattern and started on GUAUG (Supplementary Table S2). In contrast, none of the cupuladriid introns started on the conserved GUGYG motif. As a result, intron starts were determined by the end of the preceding exon, which resulted in highly variable putative start motifs (Supplementary Table S2). Similarly, the ends of introns were determined by the start of the following exons, which meant that 16 out of the 35 cupuladriid introns did not terminate on a conserved AY motif (Supplementary Table S2). The lack of these conserved motifs suggests that the cupuladriid introns might splice via alternative 5’ and 3’ splice sites (see^14^; Fig. 3I). Most Group II introns with alternative splice sites contain LAGLIDADG homing endonucleases^14^, but these could not be found in the cupuladriid mitogenomes using Pfam searches.

**Figure 4:**
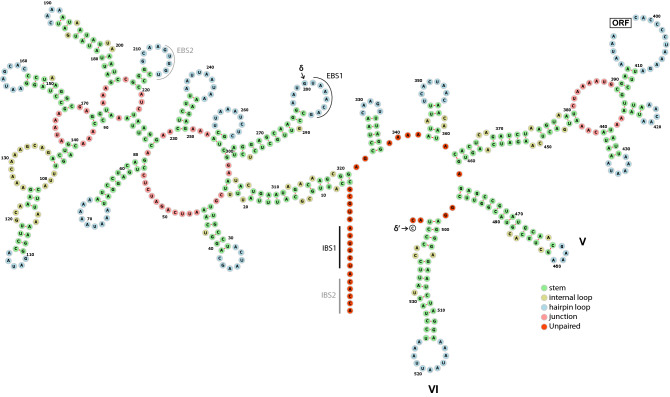
Putative secondary structure drawing of EV-cox1 intron (*Exechonella vieirai*). The intron-encoded protein open-reading frame (ORF) was excised prior to folding. Domains V and VI and intron-binding site IBS1 and corresponding exon-binding site EBS1 are indicated. Less certain IBS2 and EBS2 sites are indicated in grey. Tertiary interaction sites delta (δ) and delta prime (δ’) are labelled. Nucleotides are coloured according to their positions in the secondary structure.

Concerning the identification of intron types in our study species, we consider the introns of *E. vieirai* and *P. penelope* to be possible Group II introns due to the presence of a) IEP with RVT-IM domains, b) conserved Group II ribozyme domains V and VI, c) Group II intron-typical start and stop motifs (except the non-standard start motif in PP-cox1-ii). All introns in these two species finish on YAY, which is a typical Group IIA end motif, versus RAY, which is typical for Group IIB introns^13^. However, the secondary structures of the two IEP-containing introns (EV-cox1, PP-cox1-i; Fig. 4, Supplementary Fig. S4) do not adhere to the conserved motifs shown in the Group IIA consensus secondary structure given on the Zimmerly lab website (http://webapps2.ucalgary.ca/~groupii/)^10,49,50^. Although the secondary structure drawing of EV-cox1 (Fig. 4) shows multiple domains radiating from a central wheel, only domains V and VI could be identified reliably. Furthermore, only a few tertiary interaction features that facilitate intron–exon pairings, i.e. intron–exon binding sites IBS1-EBS1 and delta (δ) and delta prime (δ’)^51^ could be determined reliably; the IBS2-EBS2 pairing, although indicated in Fig. 4 should be treated with caution. Regarding the secondary structure of intron PP-cox1-i (Supplementary Fig. S4), only Group II domains V and VI could be reliably identified. Thus, the lack of unequivocal evidence prevents us from assigning a Group II subcategory to these introns.

Concerning *C. biporosa* and *D. cookae*, the identity of the introns cannot be ascertained because of the absence of identifiable ribozyme domains and Group II intron start/stop motifs. However, the presence of RVT-IM domains in both species indicates that they might be atypical Group II introns. Furthermore, none of the reconstructed secondary structures resemble the characteristic P1-P10 stems of Group I introns^15^ (Supplementary Figs S5, S6). Moreover, none of the Pfam searches of any of the six reading frames of the cupuladriid introns provided any hits with homing endonucleases, which are characteristic of Group I introns. Thus, we conclude that they are unlikely Group I introns. We also considered the possibility of them being Group III introns, which have been described from euglenoid chloroplast genomes^52^. Group III introns are somewhat degenerate versions of Group II introns. They are typically short (91–119 bp), AU-rich with a base bias of U > A > G > C, have degenerate Group II intron-like boundaries (5’: NUNNG; 3’: ANNUNNNN), and lack any consistent secondary structure^52–54^, although they have been shown to have a structure resembling Group II intron domain VI^55^. Regarding the cupuladriid introns, their lengths, although shorter than introns in the non-cupuladriids, exceed the typical Group III intron size. Intron sizes range from 221–519 bp (average 272 bp) and 240–397 bp (average 273 bp) in *C. biporosa* and *D. cookae*, respectively (Supplementary Tables S1, S2). Although AU rich, the most frequent nucleotide in cupuladriid introns is adenine. In *C. biporosa* the average frequencies of adenine and uracil are 53.6% and 22.6%, respectively. In *D. cookae* the average frequencies of adenine and uracil are 49.9% and 31.9%, respectively (Supplementary Table S1). Concerning intron boundaries, only one of the 18 introns in *C. biporosa* had a degenerate Group III intron 5’ end motif (NUNNG). This was found toward the 5’ end of intron CB-cox3-ii (AA**CUAAG)**. Similarly, only two of the 15 introns in *D. cookae* for which the 5’ ends were available, had the degenerate Group III intron motif towards their 5’ ends: DC-nad5-i (UGAC**UUUUG**) and DC-nad4L (**GUAUG**); nucleotides not emboldened are currently considered parts of the introns as they are the nucleotides immediately following the preceding exons. The degeneracy of the 3’ end motif means that it is present too frequently to make any meaningful inferences. Lastly, the secondary structure drawings frequently show a stem towards the 3’ end (Supplementary Figs. S5, S6) which may be a domain VI-like structure. However, at this stage, we consider the evidence for/against Group III introns too ambiguous to make any informed conclusions.

### Putative intron origin(s)

**Figure 5:**
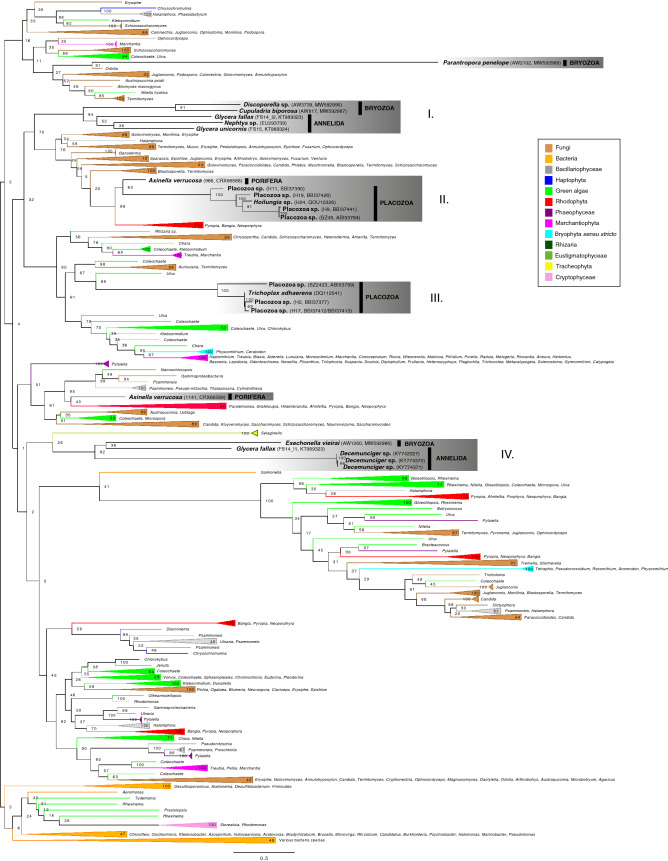
Maximum likelihood analysis of concatenated Group II reverse transcriptase and intron maturase open reading frames of *Exechonella vieirai*, *Parantropora penelope, Discoporella cookae* and *Cupuladria biporosa* and other metazoans (Porifera, Polychaeta, Placozoa), all emboldened and highlighted by shaded boxes, together with data from across the tree of life. Higher taxonomic affinities are colour coded (see insert). Ambiguously aligned positions had been excluded using Gblocks v.91b. Analysis was done using RAxML v.8.2.12 under the LG + G4 + F model with fast bootstrap analysis (1000 replicates). Values at nodes indicate maximum likelihood bootstrap values. Branch length scale bar indicates number of substitutions per site. *Clades I–IV*, as referred to in the text, are labelled.

In order to examine the origin(s) of bryozoan mitochondrial introns in an evolutionary context, a phylogeny of RVT-IM domains with representatives from across the tree of life was constructed. The following description of phylogenetic interrelationships of RVT-IM domains (Fig. 5; for ML tree with full terminal names, see Supplementary Fig. S7) focusses on the placement of the four bryozoan target taxa as well as other metazoans for which Group II IEPs with RVT-IM domains are known, i.e. Placozoa, Porifera and Annelida (polychaetes). Monophyletic groups of metazoan ORFs are indicated as *Clades I–IV.*

#### Clades I and II

The common origin of RVT-IM domains in the two cupuladriids *Cupuladria biporosa* and *Discoporella cookae* is strongly supported (91% bootstrap support [bs]). They formed strongly supported *Clade I* together with RVT-IM domains from polychaete taxa *Glycera unicornis* (FS15 isolate;^31^), *Glycera fallax* (FS14 isolate, I2 copy;^31^) and *Nephtys* sp. (94% bs); this close association of annelid terminals and the non-monophyly of the two tandemly repeated *Glycera fallax* FS14 intron copies I1 and I2 (see *Clade IV* description below for copy I1 position) was also found by Richter et al*.*^31^. This clade was sister to a paraphyletic assemblage of fungal sequences that also included the diatom *Halamphora calidilacuna*, and a strongly supported clade (99% bs) composed of RVT-IM domains of rhodophyte genera *Pyropia*, *Bangia* and *Neoporphyra* and the sponge *Axinella verrucosa* (copy 966^20^), which formed the moderately supported sister (63% bs) to a clade of five placozoan sequences (Placozoa sp. haplotypes H9, H11, H19; *Hoilungia* sp. haplotype H24^26^; Placozoa sp. strain BZ49^24^). As in Signorovitch et al*.*^24^ and Huchon et al.^20^, an independent origin of *Placozoa* sp. strains BZ49 (here together with strains H9, H11, H19 and H24) and BZ2423 + *Trichoplax adhaerens* (here together with strain H2 and H17; see *Clade III* description) was found in our analysis. Furthermore, this close association of the abovementioned rhodophyte genera *Bangia* and *Pyropia*, placozoans and sponges is in agreement with findings by Huchon et al.^20^.

#### Clade III

A second clade of placozoan sequences composed of Placozoa sp. haplotypes H2, H17^26^, Placozoa sp. strain BZ2423^24^ and *Trichoplax adhaerens*^23^ nested in a moderately supported clade (67% bs) with charophyte *Coleochaete*, chlorophyte *Ulva* and fungi *Auricularia* and *Termitomyces*. In the wider context of a well-supported node (80% bs) that included members of Rhizaria*,* Fungi, Chlorophyta, Charophyta, Marchantiophyta and Bryophyta as well as placozoan *Clade III*, our results, although using a more elaborate taxon sampling, broadly agrees with results obtained by Huchon et al*.*^20^. All genera recovered as close relatives of *Clade III* in Huchon et al*.*^20^, i.e. *Marchantia*, *Treubia*, *Klebsormidium*, *Chlorokybus* and *Schizosaccharomyces* were also found to be part of this clade. A close association (although not strongly supported) of *Trichoplax adhaerens* with *Marchantia*, *Chlorokybus* and *Schizosaccharomyces* was also found by Vallès et al*.*^30^. A novel finding of the present study, as a result of a broader taxon sampling, is the strongly supported (89%) sister-group relationship of placozoan *Clade III* with *Ulva compressa*.

#### Clade IV

*Exechonella vieirai* was placed with strong nodal support (82% bs) in a clade with polychaete taxa *Glycera fallax* (FS14 isolate, I1 copy^31^) and *Decemunciger* sp. The sister to *Clade IV* was formed by the tracheophyte *Selaginella*, but support for this was weak (26% bs). Similarly, neither Richter et al*.*^31^ nor Bernardino et al*.*^32^ were able to determine a supported position for *Glycera fallax* (FS14 isolate, I1 copy), although it formed a weakly supported clade with RVT-IM domains from *Marchantia* and land plants (*Arabidopsis*, *Solanum*, *Zea*, *Triticum*, *Vicia*, *Glycine*, *Oenothera*) in both of their analyses. Considering that both used the alignment by Zimmerly et al*.*^56^ as a starting point (versus our de novo alignment which, amongst other more recently generated sequences, included *Selaginella* [XP_024524942, XP_024518366; published in 2018]), this is to be expected. In contrast to the present analysis, in Bernardino et al*.*^32^ the RVT-IM copies of *Decemunciger* sp. formed a strongly supported clade with *Nephtys* sp. (91% bs), nesting in a weakly supported clade with *Glycera unicornis* (FS15 isolate^31^) and *Glycera fallax* (FS14 isolate, I2 copy^31^). This difference in topology might be explained by the high sequence divergence in *Decemunciger* sp. copies, as evidenced by the long branch leading to the clade of the three *Decemunciger* sp. copies in Bernardino et al*.*^32^ and in Fig. 5 in the present study. This high sequence divergence makes an unambiguous alignment difficult and, combined with a different taxon sampling, including the closely related *Exechonella vieirai* copy, might have led to this difference in topology.

In addition to *Clades I-IV*, there were two terminals that did not group with any other metazoan RVT-IM copies. The first, *Axinella verrucosa* (copy 1141^20^), formed a weakly supported clade (40% bs) with rhodophyte genera *Hildenbrandia*, *Ahnfeltia*, *Pyropia*, *Bangia*, *Neoporphyra* (as *Porphyra haitanensis* in Huchon et al*.*^20^), all of which also formed the sister group in Huchon et al*.*^20^. In addition, in the present analysis, this rhodophyte clade also included *Paralemanea*, *Grateloupia*. This *A. verrucosa* + Rhodophyta clade formed the sister group with strong support (95% bs) to a strongly supported clade (99% bs) composed of eustigmatophycean *Nannochloropsis*, a gamma-proteobacterium, and diatom genera *Psammoneis, Pseudo-nitschia*, *Thalassiosira* and *Cylindrotheca*; in Huchon et al*.*^20^ this corresponding sister group was formed of *Thalassiosira* and *Chattonella*. Thus, there is broad agreement with our analysis and that by Huchon et al*.*^20^, except that our broader taxon sampling added additional genera. We also observed, judging by the associated taxa in our analysis and that by Huchon et al*.*^20^, the two intron copies of *Axinella verrucosa* (copy 966 = GenBank accession CRX66588; copy 1141 = GenBank accession CRX66589) came out in switched positions in ours and their topologies. We suspect that this could either be due to a mislabelling of Fig. 7 in Huchon et al*.*^20^ or a mislabelling of their GenBank accession records. The position of the second terminal, *Parantropora penelope*, could not be unambiguously resolved as it nested on a very long branch in a poorly supported clade (16% bs) together with representatives of Fungi, Haptophyta, Bacillariophyceae, Chlorophyta, Charophyta and Marchantiophyta.

Our results show that Group II IEPs with RVT-IM domains were acquired independently numerous times amongst metazoans. Regarding our target bryozoan species, RVT-IM domains containing IEPs were likely acquired independently in *Exechonella vieirai* and *Parantropora penelope*. Furthermore, we infer a separate but possibly shared origin in the two cupuladriid taxa *Cupuladria biporosa* and *Discoporella cookae* (Fig. 5). Although it is conceivable that RVT-IM containing IEPs were acquired from multiple source organisms in a common ancestor of cheilostome bryozoans and were purged differentially during their evolution leading to today’s distribution of copies, the more parsimonious solution is likely the independent acquisition of introns. This question lends itself to be examined further using ancestral character estimation in the context of well sampled phylogenies which are being produced (^57^; Jenkins & Graham et al., in preparation). Moreover, the monophyly of bryozoan and annelid RVT-IM domains (*Clade I* and *Clade IV*; Fig. 5) implies that these copies had a common evolutionary origin. Much uncertainty remains regarding the insertion and propagation mechanisms of metazoan mt introns. Vertical transmission followed by independent losses has been proposed as mechanism in cnidarians and sponges^17^, whereas others have proposed a mixture of both horizontal gene transfer (HGT) and vertical transmission^18,19,58^. However, much evidence points to intron insertion via HGT from microbial or algal donors. In sponges, putative intron donors include fungi^16,21,22^, rhodophytes, diatoms and raphidophytes^20^. Additionally, placozoans have been proposed as possible intron donors in sponges^20,21^. Our analysis confirmed a close association of RVT-IM domains in sponges and placozoans (*Clade II*), as well as a close relationship of both with red algae (Fig. 5). Furthermore, *Clades I* and *II* formed a larger clade with fungal representatives, thus our findings corroborate previous speculations about possible origins. However, pinpointing the exact sources remains difficult, especially in the case of RVT-IM domains in *Axinella verrucosa* copy 1141, whose closest relative ranged from rhodophytes, diatoms, bacteria and Eustigmatophyceae. Still, the fact that we recovered four clades, each composed of multiple species and, in the cases of *Clades I*, *II* and *IV*, multiple phyla, indicates that introns in each of those clades likely originated from one type of marine organism, suggesting that certain intron donors are particularly successful in penetrating metazoan tissues.

A commonality of the metazoan taxa harbouring Group II introns is their ability to bud and regenerate. The idea that budding and regeneration ability may favour intron transmission via somatic cells/tissues was already formulated by Szitenberg et al*.*^18^ in the context of sponges. In the case of the regenerative annelid *Nephtys* sp.,^59^, Vallès et al.^30^ hypothesised that introns may have entered the germ line following HGT from possible bacterial donors via undifferentiated cells. Evidence from bryozoans in the present paper further supports the idea that organisms with regenerative abilities are easy targets for intron donors. The two cupuladriid species were found to possess an unprecedented large number of introns (18 in *Cupuladria biporosa*; 17 in *Discoporella cookae*) which is consistent with their particular reproductive life history strategy. The cupuladriid family are all free-living bryozoans that rely heavily on clonal propagation by fragmentation and regeneration of their disc-shaped colonies (e.g.^46^). This mode of reproduction often results in zooids being split open^60^ which could provide intron donors easy access to undifferentiated somatic cells. *Discoporella cookae* has undergone rates of clonal propagation exceeding 95% for at least 8 million years^61^ and clonal propagation in free-living bryozoans extends into the Cretaceous^62^. More generally, bryozoans are a rich source of bioactive compounds, many of which are likely produced by microbial symbionts^63,64^. Although the associated microbial species are mostly unknown, detailed studies on some bryozoan species have shown their colonies to harbour symbiotic bacteria on the surfaces of rhizoids^65^, intercellularly in the pallial sinus of their larvae and across larval surfaces^65,66^, as well as in tissue strands (funicular cords) that connect colony modules to one another^65,67^. Furthermore, bryozoans are often associated with microbial films^68^ and have been found to be infected by fungal species^69^. These close relationships between bryozoan hosts and microbes may have facilitated the acquisition of introns in this group.

### Within species intron relationships

**Figure 6:**
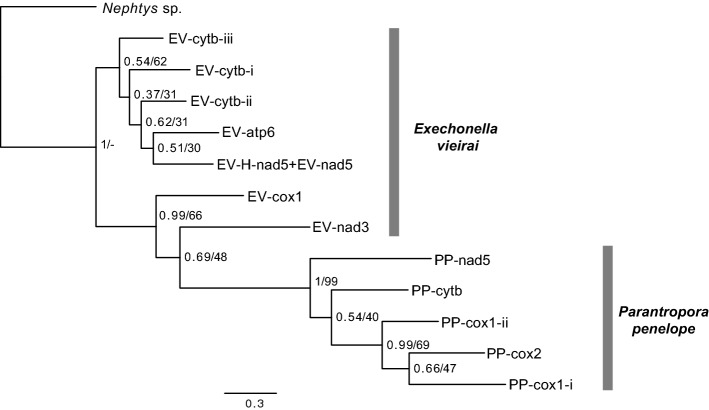
Bayesian analysis of *Exechonella vieirai* and *Parantropora penelope* intron sequences constructed using MrBayes v.3.2.6; 5,000,000 generations; 2,500,000 generations burn-in. Introns EV-H-nad5 and EV-nad5 had been concatenated. Ambiguously aligned positions had been excluded using Gblocks v.91b. Posterior probabilities and maximum likelihood bootstrap values (1000 replicates) as estimated using RAxML v.8.2.12 are given at the nodes. Analyses were carried out under the HKY + G model of nucleotide evolution. Reverse transcriptase and intron maturase open reading frames had been removed from EV-cox1 and PP-cox1-i introns. Intron names correspond to those shown in Fig. 2. Branch length scale bar indicates number of substitutions per site.

Phylogenetic analysis of IEP-less Group II introns from *E. vieirai* and *P. penelope* showed that the five copies of the latter formed a monophyly with strong nodal support (Fig. 6). Regarding the *E. vieirai* copies, two of them formed a clade with the *P. penelope* copies. However, their relationships were unresolved due to the low nodal support for one of the nodes (0.69 posterior probability, 48% bs). Thus, there is only evidence for a species-specific common ancestry of IEP-less Group II introns in *P. penelope*. This suggests that these introns may have either been inserted multiple times from the same type of source organism or that the introns self-propagated within the mitogenomes post initial insertion. Conversely, phylogenetic analyses of the IEP-less introns of the two cupuladriid taxa was completely unresolved and showed no grouping by species (Supplementary Fig. S8). Whether this is an indication of multiple insertion events from different sources or of a high post-insertion mutation rate cannot be inferred. In any case, intraspecific sequence divergence (uncorrected p-distances) was very high in all species and ranged from 38 to 60% in *E. vieirai* and 43–53% in *P. penelope* and from 39 to 59% in *C. biporosa* and 41–58% in *D. cookae* (Supplementary Tables S5, S6).

### Unusual intron characteristics

There are several features in the bryozoan mitogenomes investigated here that distinguish them from other bilaterian and metazoan intron-harbouring mitogenomes. In the context of bilaterians, our observed intron frequency in the four species of Bryozoa is unprecedented: eight in *E. vieirai* (if considering EV-H-nad5 and EV-nad5 a separate introns) and five, 18 and 17 in the incomplete mitogenomes of *P. penelope*, *C. biporosa* and *D. cookae*, respectively. This is the largest number of introns ever recorded in bilaterians (*Nephtys* sp.—one intron^30^; *Glycera fallax—*two introns, *Glycera unicornis—*one intron^31^; *Decemunciger* sp.—three introns^32^; *Cucullaea labiate—*one intron^34,35^). Furthermore, RVT-IM domains have only ever been found in cox1 in metazoans (Placozoa^23,24,26^, Porifera^20^, Polychaeta^30,32^). Although this was also the case in our *E. vieirai* and *P. penelope* mitogenomes, open-reading frames with RVT-IM domains were found in intergenic regions in *C. biporosa* and *D. cookae*. Thus, this is the first time that these domains have been found residing outside of cox1 introns in metazoans.

As foreseen by Richter et al.^31^, increased genome sequencing has revealed more Group II introns within the Bilateria, with more likely to be uncovered in the future. Nevertheless, for now at least, the frequency of mitochondrial introns in bryozoans is exceptional when compared to other bilaterians. This provides an unparalleled opportunity for bryozoans to perhaps become not only a model for studying introns but also bilaterian mitogenome architecture overall, challenging our understanding of their function and evolution. Initial observations of intron absence versus presence in a broad phylogenetic context point towards a random acquisition process, rather than one guided by shared common ancestry (unpublished data). However, future work that dissects the process of intron gain and loss amongst closely related species ought to be explored. Furthermore, seeing that HGT from microbial donors is a likely mechanism for intron integration, studying the intraspecific distribution and variability of introns could provide interesting insights into their heritability and persistence.

## Methods

### Collection information

*Parantropora penelope* (specimen ID AW2102; Fig. 1A,B) was collected from Heron Island, Queensland, Australia by A.W. and J.S.P. in January 2018 (Great Barrier Reef Marine Parks Permit G17/40024.1). *Exechonella vieirai* (specimen ID AW1260; Fig. 1C,D) was collected from the intertidal zone of Praia de Pituba, Salvador, Bahia, Brazil in July 2017 (ICMBIO/SISBIO Permit 47108-1). *Cupuladria biporosa* (specimen ID AW817; Fig. 1E) was obtained from the Golfo de Mosquitos, Caribbean Panama by S.E.C. in August 2010 (Autoridad de los recursos Acuáticos de Panamá collecting permit #DGOMI-P|CFC-N’024). *Discoporella cookae* (specimen ID AW3739; Fig. 1F) was collected around San José Island, Las Perlas archipelago, Pacific Panamá by A.O. in February 2012 (Autoridad de los recursos Acuáticos de Panamá collecting permit #DGOMI-P|CFC-N'02-A). All specimens were preserved in 95–100% ethanol. Corresponding specimens were deposited as morphological vouchers at the Natural History Museum, UK (NHMUK) collection (see Fig. 1 legend for NHMUK accession numbers). Vouchers were imaged by scanning electron microscopy using a LEO 1455-VP instrument at NHMUK.

### Illumina sequencing, assembly and annotation

Total genomic DNA (gDNA) was extracted using the DNeasy Blood & Tissue Kit (Qiagen) following manufacturer’s instructions. Double stranded (ds) DNA concentration was quantified using a Qubit™ fluorometer using either the Qubit™ dsDNA BR (Broad Range) or dsDNA HS (High Sensitivity) assay kits. Dual-indexed libraries were prepared using the TruSeq DNA Nano Library Prep Kit (Illumina, Inc., San Diego, California) for *C*. *biporosa*, and the Nextera DNA Flex Library Prep Kit (now Illumina DNA Prep; Illumina, Inc., San Diego, California) for *E. vieirai*, *P. penelope* and *D. cookae.* Sequencing was performed by Novogene (HK) Company Limited on the Illumina HiSeq 4000 platform (San Diego, California) using 2 × 150 bp paired-end sequencing.

Paired-end reads were trimmed using Trimmomatic Version 0.39^70^ and assembled de novo using SPAdes 3.13.0^71^ with k-mer sizes of 33, 55, 77, 99 and 127. Contigs of putative mitogenomes were identified by conducting blast searches^72^ against a local custom database of reference bryozoan mitogenomes in Geneious 11.1.4 (https://www.geneious.com). Candidate mt contigs were further verified by blastn searches against the NCBI database (https://blast.ncbi.nlm.nih.gov).

Mitogenome contigs were annotated using MITOS (http://mitos.bioinf.uni-leipzig.de/)^73^. PCG boundaries (start/stop codons) were refined manually in Geneious following alignment with curated sets of reference bryozoan sequences using TranslatorX (http://translatorx.co.uk/)^74^. Mitogenomes were circularised where possible using the Repeat Finder plug-in in Geneious. Assembly quality and read coverage were assessed by reference mapping of trimmed reads to mitogenome contigs under strict settings in Geneious. The settings used were as follows: allow gaps = maximum 1% per read, maximum gap size = 3, minimum overlap = 50, minimum overlap identity = 95%, word length = 18, index word length = 13, ignore words repeated more than 12 times, maximum mismatches per read 2%, maximum ambiguity = 4.

### Intron identification and validation

Introns were identified through the observation of fragmented exons. Exons were knitted together in light of alignments with non-intron containing reference data. In cases where exons had not been identified by MITOS, nucleotide data in between exons were translated into amino acids using EMBOSS Transeq (https://www.ebi.ac.uk/Tools/st/emboss_transeq) and searched using blastp (https://blast.ncbi.nlm.nih.gov) to identify additional exons. In cases where blastp failed to identify matches, the amino acid translation was scanned for conserved motifs as gleaned from reference alignments.

In order to identify intron-encoded RVT-IM domains, intron sequences were translated into amino acids using EMBOSS Transeq from all six reading frames. Translated sequences were subjected to searches using Pfam 33.1 (http://pfam.xfam.org/^75^) and blastp. No RVT-IM domains were initially found for *Cupuladria* and *Discoporella*. However, blastx searches of the complete mt fragments against the ucalgary.ca database (http://webapps2.ucalgary.ca/~groupii/cgi-bin/main/blastusr.php^50^) identified intergenic regions that were subsequently confirmed as RVT-IM domains by blastp and Pfam searches. Significant as well as insignificant Pfam results were considered. Conserved Group II ribozyme domains were identified using Rfam (https://rfam.xfam.org^76^). Intron boundaries were refined by searching for conserved 5’ (GUGYG) and 3’ terminals ([Y]AY)^12,13^.

The *bona fide* presence of 22 of the 48 identified introns was validated by PCR and Sanger sequencing, using specific exon–intron primers (Supplementary Table S4). PCR amplification used puRE Taq Ready-to-go PCR beads (Amersham Biosciences) with a total reaction volume of 25 μl, using 1 μl of 10 μM of each primer and 3 μl of template gDNA. PCR cycling conditions were as follows: initial denaturation at 94 °C for 3 min, followed by 35 cycles at 94 °C for 30 s, T_ann_ for 30 s, 72 °C for 1 min (3 min if product > 1000 bp), with a final extension step at 72 °C for 10 min (see Supplementary Table S4 for primer pair-specific T_ann_). Two PCRs (EV-cox1 and PP-cox1-i) failed and were repeated with Takara Long-range PCR kit (Takara Bio Inc.). Total reaction volume was 50 μl, using 0.5 μl enzyme, 5 μl buffer, 8 μl dNTPS, 2 μl of 10 μM of each primer and 4 μl gDNA. PCR cycling conditions were as follows: initial denaturation at 94 °C for 2 min, followed by 40 cycles at 94 °C for 20 s, 51 °C for 30 s, 68 °C for 3 min, with a final extension at 68 °C for 10 min. Sequencing using the PCR primers was performed using an Applied Biosystems 3730 DNA Analyser, using BigDye version 3.1. Sanger reads were edited in Geneious. Sequence identity was verified by mapping to the reference contigs in Geneious.

### Secondary structure drawings

Following the excision of IEP ORFs, intron sequences of *E. vieirai* and *P. penelope* were subjected to secondary structure folding at 20 °C using mfold (www.unafold.org)^77–79^. The consensus Group II secondary structures on the Zimmerly website (http://webapps2.ucalgary.ca/~groupii/)^10,49,50^ were taken as guides for subsequent manual secondary structure manipulation using forna (http://rna.tbi.univie.ac.at/forna/)^80^ in conjunction with iterative folding of parts of the sequences in mfold. Regarding intron sequences of *C. biporosa* and *D. cookae*, secondary structure folding was conducted at 20 °C using mfold, but no manual manipulations were performed.

### Intron phylogenies

Phylogenetic analyses of Group II intron RVT-IM domains were carried out as follows: Genbank searches of the protein database were conducted using terms ‘reverse transcriptase AND mitochondrion’ (2322 hits; accessed 9th March 2021) and ‘maturase AND mitochondrion’ (5187 hits; accessed 9th March 2021). Furthermore, RVT-IM domains of the following metazoans were added: *Trichoplax adhaerens* (DQ112541), Placozoa sp. (ABI53784, strain BZ49; ABI53799, strain BZ2423; BBI37377, haplotype H2; BBI37412/BBI37413, haplotype H17; BBI37390, haplotype H11; BBI37426, haplotype H19; BBI37441, haplotype H9), *Hoilungia* sp. (QOU12328, haplotype H24), *Axinella verrucosa* (CRX66588, intron 966; CRX66589, intron 1141), *Nephtys* sp. (EU293739), *Glycera unicornis* (KT989324, isolate FS15), *Glycera fallax* (KT989323, isolate FS14, introns I1 and I2) and *Decemunciger* sp. (KY742027, KY774370, KY774371); this is the first time that RVT-IM domains of placozoan haplotypes H2, H9, H11, H17, H19 and of the previously unsampled genus *Hoilungia* (H24)^26^, have been put into a ‘tree-of-life’ phylogenetic context. These ORFs were trimmed following Pfam analyses. No Pfam match for RVT-IM could be obtained for *Endomyzostoma* sp. In order to maximise our chances of identifying metazoan Group II intron origins, we also included blast matches of the abovementioned accessions and the bryozoans RVT-IM domains in our analyses. Duplicate accessions were removed in Mesquite v.3.51^81^. Amino acid sequence duplicates were removed using the web server for FASTA tools unique sequences (https://www.ncbi.nlm.nih.gov/CBBresearch/Spouge/html_ncbi/html/fasta/uniqueseq.cgi).

Initial alignments were constructed in MAFFT v.7.453^82^ using the --auto setting. Alignments were examined by eye in Geneious and obvious outliers were removed. Initial neighbour-joining trees, produced in PAUP* v.4.0a^83^, identified a large clade composed of plant RVT-IM sequences. As none of the focus sequences were nesting in this clade, it was removed from further analyses. The final alignment was carried out using the MAFFT settings --amino --bl 30 --genafpair --maxiterate 100. Ambiguously aligned positions were excluded using the stand-alone version of Gblocks v. 0.91b^84,85^ using the following settings: minimum number of sequences for a conserved position = lowest; minimum number of sequences or a flank position = lowest; maximum number of contiguous non-conserved positions = 10; minimum length of a block = 5; allowed gap positions = with half. The final alignment consisted of 1059 terminals and 3947 positions, of which 367 remained included. ModelTest-NG v.0.1.6^86^ was used to determine the best model of amino acid substitution. Maximum likelihood (ML) phylogenetic analysis with 1000 fast bootstrap replicates was carried out using RAxML v.8.2.12^87^ under the LG + G4 + F model. Uncorrected p-distances were calculated using PAUP* v.4.0a^83^.

To assess common ancestry of introns within species, phylogenetic analyses of (a) Group II IEP-less introns of *Exechonella* and *Parantropora*, and (b) IEP-less introns of *Cupuladria* and *Discoporella* were carried out. The outgroup for analysis (a) was the well-annotated Group II intron *Nephtys* sp. (EU293739^30^) from which the IEP ORF had been excised according to their secondary structure drawing (Fig. 1 in^30^); no suitable outgroup was available for analysis (b). Alignments were constructed in MAFFT with settings --genafpair --maxiterate 1000. Ambiguously aligned positions were excluded using the stand-alone version of Gblocks as outlined above. ModelTest-NG was used to determine the best model of nucleotide substitution. ML with 1000 fast bootstrap replicates and BI analyses were conducted in RAxML and MrBayes v.3.2.6^88^ under the HKY + G model. Two parallel MrBayes runs were performed for 5 million generations. The burn-in was defined as the point at which the average standard deviation of split frequencies was < 0.01.

## Supplementary Information

Supplementary Figure S1.

Supplementary Figure S2.

Supplementary Figure S3.

Supplementary Figure S4.

Supplementary Figure S5.

Supplementary Figure S6.

Supplementary Figure S7.

Supplementary Figure S8.

Supplementary Figure S9.

Supplementary Legends.

Supplementary Table S1.

Supplementary Table S2.

Supplementary Table S3.

Supplementary Table S4.

Supplementary Table S5.

Supplementary Table S6.

## Data Availability

For alignments, trees and analyses commands see TreeBASE: http://purl.org/phylo/treebase/phylows/study/TB2:S29524.

## References

[1] Gray MW (2012). Mitochondrial evolution. Cold Spring Harb. Perspect. Biol..

[2] Gray MW (1998). Genome structure and gene content in protist mitochondrial DNAs. Nucleic Acids Res..

[3] Zardoya R (2020). Recent advances in understanding mitochondrial genome diversity. F1000.

[4] Lynch M, Koskella B, Schaack S (2006). Mutation pressure and the evolution of organelle genomic architecture. Science.

[5] Lynch M (2006). Streamlining and simplification of microbial genome architecture. Annu. Rev. Microbiol..

[6] Nielsen H, Johansen SD (2009). Group I introns moving in new directions. RNA Biol..

[7] Hausner G, Hafez M, Edgell DR (2014). Bacterial group I introns: Mobile RNA catalysts. Mob. DNA.

[8] Haugen P, Simon DM, Bhattacharya D (2005). The natural history of group I introns. Trends Genet..

[9] Nawrocki EP, Jones TA, Eddy SR (2018). Group I introns are widespread in archaea. Nucleic Acids Res..

[10] Simon DM (2008). Group II introns in Eubacteria and Archaea: ORF-less introns and new varieties. RNA.

[11] Novikova O, Belfort M (2017). Mobile group II introns as ancestral eukaryotic elements. Trends Genet..

[12] Lambowitz AM, Zimmerly S (2011). Group II introns: Mobile ribozymes that invade DNA. Cold Spring Harb. Perspect. Biol..

[13] Michel F, Umesono K, Ozeki H (1989). Comparative and functional anatomy of group II catalytic introns – A review. Gene.

[14] Zimmerly S, Semper C (2015). Evolution of group II introns. Mob. DNA.

[15] Michel F, Westhof E (1990). Modelling of the three-dimensional architecture of group I catalytic introns based on comparative sequence analysis. J. Mol. Biol..

[16] Rot C, Goldfarb I, Ilan M, Huchon D (2006). Putative cross-kingdom horizontal gene transfer in sponge (Porifera) mitochondria. BMC Evol. Biol..

[17] Wang X, Lavrov DV (2008). Seventeen new complete mtDNA sequences reveal extensive mitochondrial genome evolution within the Demospongiae. PLoS ONE.

[18] Szitenberg A, Rot C, Ilan M, Huchon D (2010). Diversity of sponge mitochondrial introns revealed by cox1 sequences of Tetillidae. BMC Evol. Biol..

[19] Erpenbeck D, Aryasari R, Hooper JNA, Wörheide G (2015). A mitochondrial intron in a verongid sponge. J. Mol. Evol..

[20] Huchon D, Szitenberg A, Shefer S, Ilan M, Feldstein T (2015). Mitochondrial group I and group II introns in the sponge orders Agelasida and Axinellida. BMC Evol. Biol..

[21] Kelly M, Cárdenas P (2016). An unprecedented new genus and family of Tetractinellida (Porifera, Demospongiae) from New Zealand’s Colville Ridge, with a new type of mitochondrial group I intron. Zoo. J. Linn. Soc.-Lond..

[22] Cranston A, Taboada S, Koutsouveli V, Schuster A, Riesgo A (2021). A population specific mitochondrial intron from the sponge *Phakellia robusta* in the North-East Atlantic. Deep-Sea Res. Pt. I.

[23] Dellaporta SL (2006). Mitochondrial genome of *Trichoplax adherens* supports Placozoa as the basal lower metazoan phylum. Proc. Natl. Acad. Sci. USA.

[24] Signorovitch AY, Buss LW, Dellaporta SL (2007). Comparative genomics of large mitochondria in placozoans. PLoS Genet..

[25] Burger G, Yan Y, Javadi P, Lang BF (2009). Group I-intron trans-splicing and mRNA editing in the mitochondria of placozoan animals. Trends Genet..

[26] Miyazawa H (2021). Mitochondrial genome evolution of placozoans: gene rearrangements and repeat expansions. Genome Biol. Evol..

[27] Beagley CT, Okada NA, Wolstenholme DR (1996). Two mitochondrial group I introns in a metazoan, the sea anemone *Metridium senile*: One intron contains genes for subunits 1 and 3 of NADH dehydrogenase. Proc. Natl. Acad. Sci. USA.

[28] Van Oppen MJH (2000). The mitochondrial genome of *Acropora tenuis* (Cnidaria; Scleractinia) contains a large group I intron and a candidate control region. J. Mol. Evol..

[29] Goddard MR, Leigh J, Roger AJ, Pemberton AJ (2006). Invasion and persistence of a selfish gene in the Cnidaria. PLoS ONE.

[30] Vallès Y, Halanynch KM, Boore JL (2008). Group II introns break new boundaries: Presence in a bilaterian’s genome. PLoS ONE.

[31] Richter S, Schwarz F, Hering L, Böggemann M, Bleidorn C (2015). The utility of genome skimming for phylogenomic analyses as demonstrated for glycerid relationships (Annelida, Glyceridae). Genome Biol. Evol..

[32] Bernardino AF, Li Y, Smith CR, Halanych KM (2017). Multiple introns in a deep-sea annelid (*Decemunciger*: Ampharetidae) mitochondrial genome. Sci. Rep..

[33] Zhong, M. Applicability of mitochondrial genome data to annelid phylogeny and the evolution of group II introns. *PhD Thesis*. (Auburn University, 2009).

[34] Feng Y, Li Q, Yu H, Kong L (2017). Complete mitochondrial genome sequence of *Cucullaea labiata* (Arcoida: Cucullaeidae) and phylogenetic implications. Genes Genom..

[35] Kong L (2020). Mitogenomics reveals phylogenetic relationships of Arcoida (Mollusca, Bivalvia) and multiple independent expansions and contractions in mitochondrial genome size. Mol. Phylogenet. Evol..

[36] Zhang Z (2021). Fossil evidence unveils an early Cambrian origin for Bryozoa. Nature.

[37] Ryland JS (1970). Bryozoa.

[38] Schwaha, T. *Handbook of Zoology: Phylum Bryozoa* (ed. Schwaha, T.) (De Gryter, 2020).

[39] Hageman SJ, Bock PE, Bone Y, McGowran B (1998). Bryozoan growth habits: Classification and analysis. J. Paleontol..

[40] Wood ACL, Probert PK, Rowden AA, Smith AM (2012). Complex habitat generated by marine bryozoans: a review of its distribution, structure, diversity, threats and conservation. Aquatic Conserv. Mar. Freshw. Ecosyst..

[41] Bock PE, Gordon DP (2013). Phylum Bryozoa Ehrenberg, 1831. Zootaxa.

[42] Waeschenbach A, Taylor PD, Littlewood DTJ (2012). A molecular phylogeny of bryozoans. Mol. Phylogenet. Evol..

[43] Bushnell JH, Rao KS (1974). Dormant or quiescent stages and structures among the Ectoprocta: physical and chemical factors affecting viability and germination of statoblasts. Trans. Am. Microsc. Soc..

[44] Lidgard S (1986). Ontogeny in animal colonies: A persistent trend in the bryozoan fossil record. Science.

[45] Taylor PD, Di Martino E, Martha SO (2019). Colony growth strategies, dormancy and repair in some Late Cretaceous encrusting bryozoans: Insights into the ecology of the Chalk seabed. Palaeobiol. Palaeoenviron..

[46] O’Dea A, Herrera-Cubilla A, Fortunato H, Jackson BC (2004). Life history variation in cupuladriid bryozoans from either side of the Isthmus of Panama. Mar. Ecol. Prog. Ser..

[47] Ojala D, Montoya J, Attardi G (1981). tRNA punctuation model of RNA processing in human mitochondria. Nature.

[48] Dubot C (2004). GUG is an efficient initiation codon to translate the human mitochondrial ATP6 gene. Biochem. Biophys. Res. Co..

[49] Dai L, Toor N, Olson R, Keeping A, Zimmerly S (2003). Database for mobile group II introns. Nucleic Acids Res..

[50] Candales MA (2012). Database for bacterial group II introns. Nucleic Acids Res..

[51] McNeil BA, Semper C, Zimmerly S (2016). Group II introns: Versatile ribozymes and retroelements. Wiley Interdiscip. Rev. RNA.

[52] Christopher DA, Hallick RB (1989). *Euglena gracilis* chloroplast ribosomal protein operon: a new chloroplast gene for ribosomal protein L5 and description of a novel organelle intron category designated group III. Nucleic Acids. Res..

[53] Copertino DW, Hallick RB (1993). Group II and group III introns of twintrons: Potential relationships with nuclear pre-mRNA introns. Trends Biochem. Sci..

[54] Hong L, Hallick RB (1994). A group III intron is formed from domains of two individual group II introns. Gene. Dev..

[55] Copertino DW, Hall ET, Van Hook FW, Jenkins KP, Hallick RB (1994). A group III twintron encoding a maturase-like gene exercises through lariat intermediates. Nucleic Acids. Res..

[56] Zimmerly S, Hausner G, Wu X-C (2001). Phylogenetic relationships among group II intron ORFs. Nucleic Acids Res..

[57] Orr R. J. S. *et al.* Paleozoic origins of cheilostome bryozoans and their parental care inferred by a new genome-skimmed phylogeny. *Sci. Adv.* **8**, eabm7452 (2022). 10.1126/sciadv.abm7452 PMC8967238 35353568

[58] Fukami H, Chen CA, Chiou C-Y, Knowlton N (2007). Novel group I introns encoding a putative homing endonuclease in the mitochondrial cox1 gene of scleractinian corals. J. Mol. Evol..

[59] Clark ME (1968). Later stages of regeneration in the polychaete. Nephtys. J. Morph..

[60] O’Dea A, Jackson JBC, Taylor PD, Rodriguez F (2008). Modes of reproduction in recent and fossil cupuladriid bryozoans. Palaeontology.

[61] O’Dea A, Jackson JBC (2009). Environmental change drove macroevolution in cupuladriid bryozoans. Proc. R. Soc. Lond. B Biol..

[62] O’Dea A, Håkansson E, Taylor PD, Okamura B (2011). Environmental change prior to the K-T boundary inferred from temporal variation in the morphology of cheilostome bryozoans. Palaeogeogr. Palaeocl..

[63] Figuerola B, Avila C (2019). The phylym Bryozoa as promising source of anticancer drugs. Mar. Drugs.

[64] Ciavatta ML (2020). The phylum Bryozoa: From biology to biomedical potential. Mar. Drugs.

[65] Sharp KH, Davidson SK, Haygood MG (2007). Localization of ‘*Candidatus* Endobugula sertula’ and the bryostatins throughout the life cycle of the bryozoan *Bugula neritina*. ISME J..

[66] Woollacott RM (1981). Association of bacteria with bryozoan larvae. Mar. Biol..

[67] Karagodina NP, Vishnyakov AE, Kotenko ON, Maltseva AL, Ostrovsky AN (2018). Ultrastructural evidence for nutritional relationships between a marine colonial invertebrate (Bryozoa) and its bacterial symbionts. Symbiosis.

[68] Scholz J (1995). Epibiontic microorganisms as a local control factor of bryozoan distribution and bryozoans ‘micro-reefs’. Beitr. Paläontol..

[69] Sterflinger K, Hain M, Scholz J, Wasson K (2001). Fungal infections of a colonial marine invertebrate: Diversity and morphological consequences. Facies.

[70] Bolger AM, Lohse M, Usadel B (2014). Trimmomatic: A flexible trimmer for Illumina sequence data. Bioinformatics.

[71] Bankevich A (2012). SPAdes: A new genome assembly algorithm and its applications to single-cell sequencing. J. Comput. Biol..

[72] Altschul SF (1990). Basic local alignment search tool. J. Mol. Biol..

[73] Bernt M (2013). MITOS: Improved de novo metazoan mitochondrial genome annotation. Mol. Phylogenet. Evol..

[74] Abascal F, Zardoya R, Telford MJ (2010). TranslatorX: Multiple alignment of nucleotide sequences guided by amino acid translations. Nucleic Acids Res..

[75] Mistry J (2021). Pfam: The protein families database in 2021. Nucleic Acids Res..

[76] Kalvari I (2018). Rfam 13.0: Shifting to a genome-centric resource for non-coding RNA families. Nucleic Acids Res..

[77] Zuker M (2003). Mfold web server for nucleic acid folding and hybridization prediction. Nucleic Acids Res..

[78] SantaLucia J (1998). A unified view of polymer, dumbbell, and oligonucleotide DNA nearest-neighbor thermodynamics. Proc. Natl. Acad. Sci. USA.

[79] Peyret N (2000). Prediction of Nucleic Acid Hybridization: Parameters and Algorithms. PhD Dissertation.

[80] Kerpedjiev P, Hammer S, Hofacker IL (2015). Forna (force-directed RNA): Simple and effective online RNA secondary structure diagrams. Bioinformatics.

[81] Maddison, W. P. & Maddison, D. R. *Mesquite: A Modular System for Evolutionary Analysis. Version 3.61*. http://www.mesquiteproject.org (2019).

[82] Katoh K, Standley DM (2013). MAFFT multiple sequence alignment software version 7: Improvements in performance and usability. Mol. Biol. Evol..

[83] Swofford, D. L. *PAUP*. Phylogenetic Analysis Using Parsimony (*and Other Methods). Version 4*. (Sinauer Associates, 2003).

[84] Castresana J (2000). Selection of conserved blocks from multiple alignments for their use in phylogenetic analysis. Mol. Biol. Evol..

[85] Talavera G, Castresana J (2007). Improvement of phylogenies after removing divergent and ambiguously aligned blocks from protein sequence alignments. Syst. Biol..

[86] Darriba D (2019). ModelTest-NG: A new and scalable tool for the selection of DNA and protein evolutionary models. Mol. Biol. Evol..

[87] Stamatakis A (2014). RAxML version 8: A tool for phylogenetic analysis and post-analysis of large phylogenies. Bioinformatics.

[88] Ronquist F (2012). MrBayes 3.2: Efficient Bayesian phylogenetic inference and model choice across a large model space. Syst. Biol..

