# Supplementary figures and images for: Unprecedented frequency of mitochondrial introns in colonial bilaterians
Source: Sci Rep. 2022 Jun 28;12:10889. doi: 10.1038/s41598-022-14477-3 (PMC9240083; doi:10.1038/s41598-022-14477-3)

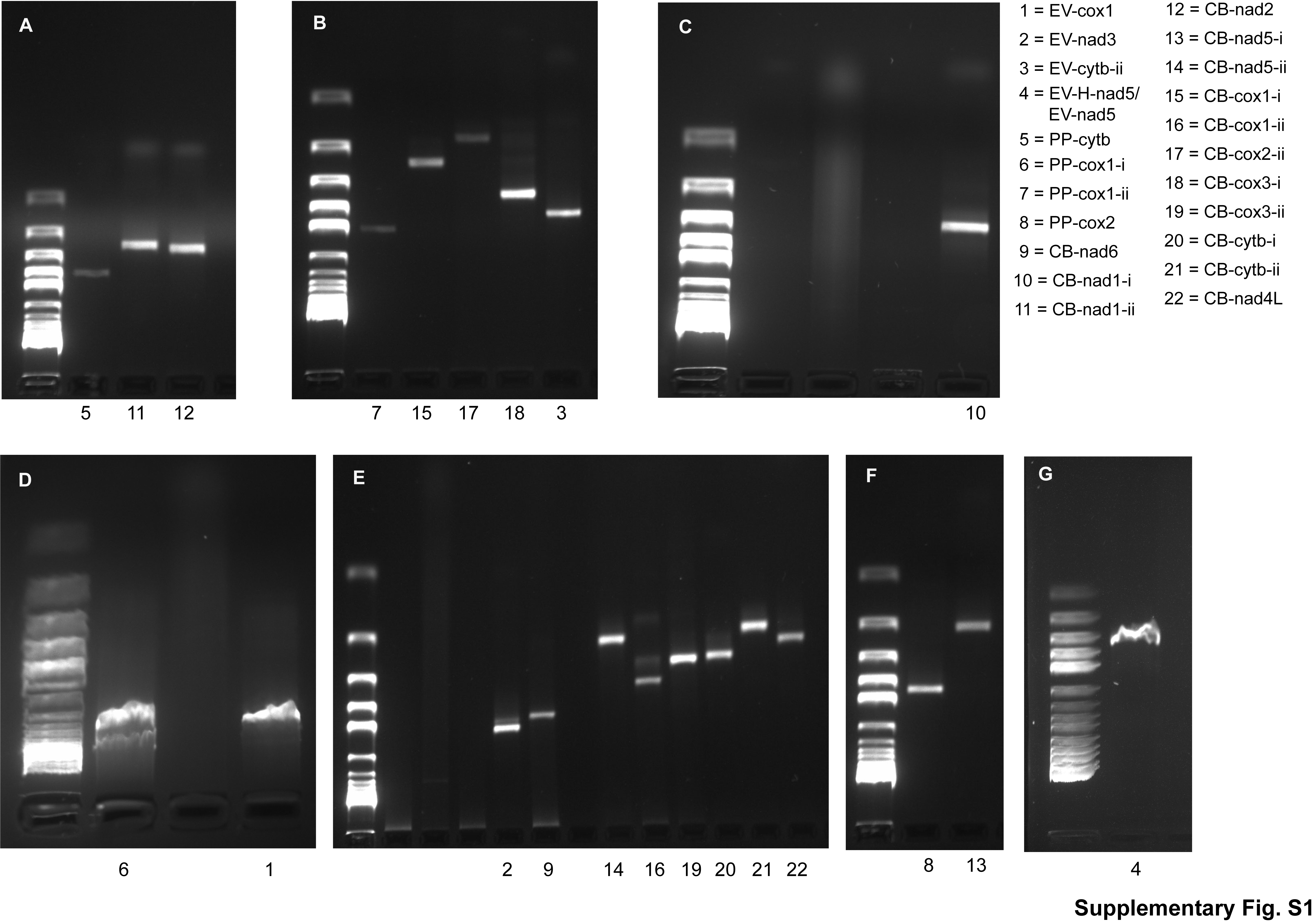

Supplement: Supplementary file 1 — Supplementary Figure S1. [file 41598_2022_14477_MOESM1_ESM.tif]
