# Supplementary material for: Unprecedented frequency of mitochondrial introns in colonial bilaterians
Source: Sci Rep. 2022 Jun 28;12:10889. doi: 10.1038/s41598-022-14477-3 (PMC9240083; doi:10.1038/s41598-022-14477-3)
Supplement: Supplementary file 10 — Supplementary Legends. [file 41598_2022_14477_MOESM10_ESM.docx]

**Supplementary Information**

Supplementary Figure S1. Cropped gel images of PCR products spanning introns. Bands correspond to the expected product sizes estimated from the Illumina read assemblies. Numbers refer to PCR products 1-22 shown in Figure 2 and listed in Supplementary Table S4. Marker: HyperLadder 1kb (Bioline). Ladder band sizes (bp): 200, 400, 600, 800, 1000, 1500, 2000, 2500, 3000, 4000, 5000, 6000, 8000, 10000. Imaging done using software GeneSys v.1.6.3.0 using a Syngene G:BOX F3-LFB. Image editing and cropping was done in Adobe Illustrator v.25.0.1. Original, unedited and uncropped gel images are presented in Supplementary Figure S9.

Supplementary Figure S2. Alignment of the 5’ end of published and unpublished bryozoan nad5 sequences. Codons are shaded according to amino acids. The orange box in the first taxon, *Exechonella vieirai*, indicates the 114 bp exon that interrupts the EV-H-nad5/EV-nad5 intron.

Supplementary Figure S3. Pfam results of reverse transcriptase and intron maturase open reading frames.

Supplementary Figure S4. Putative secondary structure drawing of PP-cox1-i intron (*Parantropora penelope*). The intron-encoded protein open-reading frame (ORF) was excised prior to folding. Domains V and VI are indicated. Nucleotides are coloured according to their positions in the secondary structure.

Supplementary Figure S5. Secondary structure drawings of *Cupuladria biporosa* introns.

Supplementary Figure S6. Secondary structure drawings of *Discoporella cookae* introns.

Supplementary Figure S7. Maximum likelihood analysis of concatenated Group II reverse transcriptase and intron maturase domains of *Exechonella vieirai*, *Parantropora penelope, Discoporella cookae* and *Cupuladria* *biporosa* and other metazoans (Porifera, Polychaeta, Placozoa) together with data from across the tree of life. Ambiguously aligned positions had been excluded using Gblocks v.91b. Analysis was done using RAxML v.8.2.12 under the LG+G4+F model with fast bootstrap analysis (1000 replicates). Values at nodes indicate maximum likelihood bootstrap values. Branch length scale bar indicates number of substitutions per site. GenBank accession numbers are given as part of the terminal names.

Supplementary Figure S8. Bayesian analysis of *Cupuladria* *biporosa* and *Discoporella cookae* intron sequences constructed using MrBayes v.3.2.6; 5,000,000 generations; 2,500,000 generations burn-in. Ambiguously aligned positions had been excluded using Gblocks v.91b. Posterior probabilities are given at the nodes. The analysis was carried out under the HKY+G model of nucleotide evolution. Intron names correspond to those shown in Figure 2. Branch length scale bar indicates number of substitutions per site.

Supplementary Figure S9. Uncropped, original gel images of PCR products spanning introns as shown in Supplementary Figure S1. Marker: HyperLadder 1kb (Bioline). Ladder band sizes (bp): 200, 400, 600, 800, 1000, 1500, 2000, 2500, 3000, 4000, 5000, 6000, 8000, 10000. Imaging done using softward GeneSys v.1.6.3.0 using a Syngene G:BOX F3-LFB.

Supplementary Table S1. Mitogenome information (gene boundaries, gene lengths, start and stop codons, tRNA codons, GC %, and gene direction; F = forward, R = reverse) for *Exechonella vieirai* (a), *Parantropora penelope* (b)*, Cupuladria* *biporosa* (c), and *Discoporella cookae* (d, e). A/T % are given for *C. biporosa* and *D. cookae* introns.

Supplementary Table S2. List of introns found in the mitogenomes of *Exechonella vieirai*, *Parantropora penelope*, *Cupuladria* *biporosa*, and *Discoporella cookae*. Capital prefixes of intron names represent genus/species initials. Whenever multiple introns were found per gene, introns are labelled with suffixes i-iii. Intron-encoded proteins (IEPs) with reverse transcriptase (RVT) and intron maturase (IM) domains are indicated. *Intron putatively interrupted by short nad5 ORF. **5’ end missing. ^‡^Intron verified by PCR. Standard start (GUGYG) and stop ([Y]AY) motifs are indicated in bold.

Supplementary Table S3. Rfam results for *Exechonella vieirai* and *Parantropora penelope*.

Supplementary Table S4. Specific primers designed and used in this study to confirm the presence of introns. PCR product sizes are given in ‘Size (bp)’. ‘T_ann_ (°C)’ are annealing temperatures used in PCR cycling, * denotes long-range PCRs. PCR numbers correspond to those shown in Figure 2 and Supplementary Figure S1.

Supplementary Table S5. Uncorrected p-distances of *Exechonella vieirai* and *Parantropora penelope* introns. Intron-encoded protein sequences had been excised from AW1260 EV-cox1 and AW2102 PP-cox1-i.

Supplementary Table S6. Uncorrected p-distances of *Cupuladria biporosa* and *Discoporella cookae*.
