# Supplementary material for: Unprecedented frequency of mitochondrial introns in colonial bilaterians
Source: Sci Rep. 2022 Jun 28;12:10889. doi: 10.1038/s41598-022-14477-3 (PMC9240083; doi:10.1038/s41598-022-14477-3)
Supplement: Supplementary file 11 — Supplementary Table S1. [file 41598_2022_14477_MOESM11_ESM.docx]

**Supplementary Table S1**. Mitogenome information (gene boundaries, gene lengths, start and stop codons, tRNA codons, GC %, and gene direction; F = forward, R = reverse) for *Exechonella vieirai* (a), *Parantropora penelope* (b)*, Cupuladria biporosa* (c), and *Discoporella cookae*. (d, e). A/T % are given for *C. biporosa* and *D. cookae* introns.

| 1. ***Exechonella vieirai* (AW1260) 23,057 bp (complete); GenBank acc. MW592986** | | | | | | | | |
| --- | --- | --- | --- | --- | --- | --- | --- | --- |
| **Gene** | **From** | **To** | **Size (bp)** | **Start codon** | **Stop codon** | **tRNA**  **codon** | **GC (%)** | **F/R** |
| cox1 CDS1 | 1 | 894 | 894 | AUA |  |  | 40.4 | F |
| Intron EV-cox1 | 893 | 2974 | 2082 |  |  |  | 31.1 |  |
| Intron-encoded protein | 1290 | 2828 | 1539 | AUG | UAA |  | 29.0 | F |
| Group II reverse transcriptase | <1689 | >2393 | 705 | ? | ? |  | 27.9 | F |
| Group II intron maturase | <2427 | >2768 | 342 | ? | ? |  | 31.6 | F |
| cox1 CDS2 | 2977 | 3645 | 669 |  | UAA |  | 39.9 | F |
| trnD | 3667 | 3731 | 65 |  |  | GAC | 29.2 | F |
| atp8 | 3757 | 3855 | 99 | AUG | UAA |  | 25.3 | F |
| trnF | 3897 | 3966 | 70 |  |  | TTC | 34.3 | F |
| nad3 CDS1 | 3973 | 4158 | 186 | AUG |  |  | 37.6 | F |
| Intron EV-nad3 | 4163 | 4986 | 824 |  |  |  | 37.7 |  |
| nad3 CDS2 | 4983 | 5150 | 168 |  | UAA |  | 29.8 | F |
| trnT | 5193 | 5258 | 66 |  |  | ACA | 45.5 | F |
| nad2 | 5268 | 6224 | 957 | AUG | UAA |  | 32.7 | F |
| cox2 | 6254 | 6919 | 666 | AUG | UAA |  | 36.3 | F |
| trnY | 6929 | 6989 | 61 |  |  | TAC | 36.1 | F |
| trnC | 6990 | 7058 | 69 |  |  | TGC | 31.9 | F |
| trnS1 | 7077 | 7137 | 61 |  |  | AGA | 31.1 | F |
| cytb CDS1 | 7184 | 7246 | 63 | AUG |  |  | 23.8 | F |
| Intron EV-cytb-i | 7247 | 7827 | 581 |  |  |  | 34.3 |  |
| cytb CDS2 | 7828 | 8472 | 645 |  |  |  | 38.3 | F |
| Intron EV-cytb-ii | 8472 | 8998 | 527 |  |  |  | 34.0 |  |
| cytb CDS3 | 9000 | 9302 | 303 |  |  |  | 38.9 | F |
| Intron EV-cytb-iii | 9302 | 9883 | 582 |  |  |  | 36.1 |  |
| cytb CDS4 | 9885 | 10010 | 126 |  | UAA |  | 33.3 | F |
| trnR | 10009 | 10077 | 69 |  |  | CGA | 20.3 | F |
| nad4L | 10081 | 10383 | 303 | AUG | UAA |  | 35.3 | F |
| nad4 | 10398 | 11736 | 1339 | AUA | U* |  | 31.8 | F |
| trnH | 11737 | 11799 | 63 |  |  | CAC | 20.6 | F |
| Intron EV-H-nad5 | 11916 | 13453 | 1538 |  |  |  | 31.7 |  |
| nad5 CDS1 | 13454 | 13567 | 114 | AUA |  |  | 29.8 | F |
| Intron EV-nad5 | 13568 | 13833 | 266 |  |  |  | 29.7 |  |
| nad5 CDS2 | 13838 | 15421 | 1584 |  | UAA |  | 34.8 | F |
| trnW | 15427 | 15494 | 68 |  |  | TGA | 23.5 | F |
| trnN | 15496 | 15562 | 67 |  |  | AAC | 35.8 | F |
| trnA | 15563 | 15629 | 67 |  |  | GCA | 40.3 | F |
| trnK | 15635 | 15704 | 70 |  |  | AAA | 34.3 | F |
| trnE | 15818 | 15883 | 66 |  |  | GAA | 36.4 | F |
| trnQ | 15934 | 15999 | 66 |  |  | CAA | 37.9 | F |
| atp6 CDS1 | 16007 | 16489 | 483 | AUG |  |  | 33.7 | F |
| Intron EV-atp6 | 16489 | 17094 | 606 |  |  |  | 34.2 |  |
| atp6 CDS2 | 17096 | 17314 | 219 |  | UAA |  | 37.0 | F |
| nad6 | 17338 | 17793 | 456 | AUA | UAA |  | 33.1 | F |
| trnM | 17792 | 17856 | 65 |  |  | ATG | 40.0 | F |
| rrnS | 17873 | 18707 | 835 |  |  |  | 33.4 | F |
| trnL2 | 18708 | 18770 | 63 |  |  | TTA | 22.2 | F |
| trnL1 | 18775 | 18834 | 60 |  |  | CTA | 31.7 | F |
| cox3 | 18841 | 19653 | 813 | AUG | UAA |  | 41.3 | F |
| nad1 | 19677 | 20598 | 922 | AUA | U* |  | 35.5 | F |
| trnG | 20602 | 20666 | 65 |  |  | GGA | 30.8 | F |
| trnV | 20680 | 20743 | 64 |  |  | GTA | 29.7 | F |
| trnI | 20766 | 20834 | 69 |  |  | ATC | 40.6 | F |
| rrnL | 21557 | 22910 | 1354 |  |  |  | 31.7 | F |
| trnP | 22911 | 22977 | 67 |  |  | CCA | 29.9 | F |
| trnS2 | 22995 | 23053 | 59 |  |  | TCA | 23.7 | F |

| 1. ***Parantropora penelope* (AW2102) 21,889 bp (partial); GenBank acc. MW592988** | | | | | | | | |
| --- | --- | --- | --- | --- | --- | --- | --- | --- |
| **Gene** | **From** | **To** | **Size (bp)** | **Start codon** | **Stop codon** | **tRNA**  **codon** | **GC (%)** | **F/R** |
| nad1 | <1 | 831 | 831 | ? | UAA |  | 35.1 | F |
| trnL1 | 849 | 916 | 68 |  |  | CTA | 23.5 | F |
| trnC | 941 | 1007 | 67 |  |  | TGC | 34.3 | F |
| trnG | 1073 | 1138 | 66 |  |  | GGA | 30.3 | F |
| trnY | 1159 | 1216 | 58 |  |  | TAC | 43.1 | F |
| trnI | 1236 | 1303 | 68 |  |  | ATC | 30.9 | F |
| trnV | 1324 | 1381 | 58 |  |  | GTA | 31.0 | F |
| nad6 | 1395 | 1859 | 465 | GUG | UAG |  | 32.3 | F |
| trnN | 3448 | 3517 | 70 |  |  | AAC | 38.6 | F |
| cytb CDS1 | 3823 | 4233 | 411 | AUG |  |  | 35.3 | F |
| Intron PP-cytb | 4235 | 4858 | 624 |  |  |  | 29.8 |  |
| cytb CDS2 | 4858 | 5583 | 726 |  | UAA |  | 33.1 | F |
| trnR | 5583 | 5650 | 68 |  |  | CGA | 32.4 | F |
| nad4L | 5657 | 5962 | 306 | GUU^§^ | UAG |  | 28.4 | F |
| nad4 | 5971 | 7305 | 1335 | AUU | UAG |  | 32.2 | F |
| trnH | 7305 | 7372 | 68 |  |  | CAC | 26.5 | F |
| nad5 CDS1 | 7350 | 7406 | 57 | AUA |  |  | 36.8 | F |
| Intron PP-nad5 | 7407 | 8038 | 632 |  |  |  | 29.4 |  |
| nad5 CDS2 | 8040 | 9647 | 1608 |  | UAA |  | 30.7 | F |
| trnQ | 9653 | 9713 | 61 |  |  | CAA | 32.8 | F |
| atp6 | 9726 | 10397 | 672 | AUU | UAA |  | 33.2 | F |
| trnK | 10483 | 10549 | 67 |  |  | AAA | 37.3 | F |
| cox1 CDS1 | 10577 | 10753 | 177 | AUA |  |  | 36.7 | F |
| Intron PP-cox1-i | 10754 | 12963 | 2210 |  |  |  | 30.7 |  |
| Intron-encoded protein | 11242 | 12810 | 1569 | AUA | UAG |  | 31.2 | F |
| Group II reverse transcriptase | <11605 | >12033 | 429 | ? | ? |  | 35.0 | F |
| Group II intron maturase | <12439 | >12726 | 288 | ? | ? |  | 28.4 | F |
| cox1 CDS2 | 12964 | 14037 | 1074 |  |  |  | 36.6 | F |
| Intron PP-cox1-ii | 14038 | 14564 | 527 |  |  |  | 29.4 |  |
| cox1 CDS3 | 14565 | 14861 | 297 |  | UAA |  | 33.3 | F |
| atp8 | 14916 | 15014 | 99 | AUG | UAG |  | 33.3 | F |
| trnF | 15028 | 15093 | 66 |  |  | TTC | 34.8 | F |
| nad3 | 15093 | 15455 | 363 | AUA | UAA |  | 32.0 | F |
| trnT | 15463 | 15530 | 68 |  |  | ACA | 35.3 | F |
| nad2 | 15547 | 16482 | 936 | AUA | UAA |  | 30.4 | F |
| cox2 CDS1 | 16488 | 16583 | 96 | AUG |  |  | 30.2 | F |
| Intron PP-cox2 | 16581 | 17212 | 632 |  |  |  | 32.4 |  |
| cox2 CDS2 | 17216 | 17797 | 582 |  | UAA |  | 33.5 | F |
| trnP | 17846 | 17912 | 67 |  |  | CCA | 26.9 | F |
| trnM | 18153 | 18217 | 65 |  |  | ATG | 40.0 | F |
| rrnS | 18219 | 19105 | 887 |  |  |  | 35.4 | F |
| trnS2 | 19201 | 19260 | 60 |  |  | TCA | 28.3 | F |
| trnS1 | 19291 | 19354 | 64 |  |  | AGA | 40.6 | F |
| trnD | 19368 | 19435 | 68 |  |  | GAC | 38.2 | F |
| trnL2 | 19466 | 19524 | 59 |  |  | TTA | 40.7 | F |
| trnM | 19547 | 19615 | 69 |  |  | ATG | 34.8 | F |
| rrnL | 19616 | 20946 | 1331 |  |  |  | 31.9 | F |
| cox3 | 20956 | 21768 | 813 | AUG | UAG |  | 38.1 | F |
| trnA | 21823 | 21889 | 67 |  |  | GCA | 31.3 | F |

|  | ***Cupuladria biporosa* (AW817) 23,200 bp (partial); GenBank acc. MW592987** | | | | | | | | | |
| --- | --- | --- | --- | --- | --- | --- | --- | --- | --- | --- |
| **Gene** | | **From** | **To** | **Size (bp)** | **Start codon** | **Stop codon** | **tRNA**  **codon** | **GC (%)** | **A/T (%)** | **F/R** |
| atp6 | | 1 | 690 | 690 | AUA | UAA |  | 32.5 |  | F |
| trnW | | 696 | 762 | 67 |  |  | TGA | 23.9 |  | F |
| nad6 CDS1 | | 779 | 952 | 174 | AUG |  |  | 28.2 |  | F |
| Intron CB-nad6 | | 953 | 1471 | 519 |  |  |  | 19.7 | 53.8/  26.6 |  |
| nad6 CDS2 | | 1472 | 1747 | 276 |  | UAA |  | 22.8 |  | F |
| trnH | | 2041 | 2111 | 71 |  |  | CAC | 23.9 |  | F |
| trnC | | 2114 | 2177 | 64 |  |  | TGC | 12.5 |  | F |
| nad1 CDS1 | | 2498 | 2755 | 258 | AUU |  |  | 27.9 |  | F |
| Intron CB-nad1-i | | 2756 | 3021 | 266 |  |  |  | 27.8 | 51.9/  20.3 |  |
| nad1 CDS2 | | 3022 | 3153 | 132 |  |  |  | 37.9 |  | F |
| Intron CB-nad1-ii | | 3154 | 3415 | 262 |  |  |  | 21.4 | 52.3/  26.3 |  |
| nad1 CDS3 | | 3416 | 3898 | 483 |  | UAA |  | 31.3 |  | F |
| trnN | | 4116 | 4184 | 69 |  |  | AAC | 30.4 |  | F |
| trnA | | 4210 | 4277 | 68 |  |  | GCA | 27.9 |  | F |
| trnR | | 4282 | 4345 | 64 |  |  | CGA | 21.9 |  | F |
| trnE | | 4351 | 4415 | 65 |  |  | GAA | 24.6 |  | F |
| nad2 CDS1 | | 4690 | 5073 | 384 | AUA |  |  | 29.9 |  | F |
| Intron CB-nad2 | | 5074 | 5333 | 260 |  |  |  | 18.1 | 55.4/  26.5 |  |
| nad2 CDS2 | | 5334 | 5882 | 549 |  | UAA |  | 25.7 |  | F |
| trnV | | 5887 | 5950 | 64 |  |  | GTA | 25.0 |  | F |
| nad5 CDS1 | | 5974 | 6354 | 381 | AUG |  |  | 26.0 |  | F |
| Intron CB-nad5-i | | 6355 | 6608 | 254 |  |  |  | 24.4 | 50.8/  24.8 |  |
| nad5 CDS2 | | 6609 | 6791 | 183 |  |  |  | 31.1 |  | F |
| Intron CB-nad5-ii | | 6792 | 7047 | 256 |  |  |  | 21.5 | 57,8/  20.7 |  |
| nad5 CDS3 | | 7048 | 8190 | 1143 |  | UAA |  | 30.9 |  | F |
| trnD | | 8258 | 8327 | 70 |  |  | GAC | 28.6 |  | F |
| cox1 CDS1 | | 8344 | 8553 | 210 | AUA |  |  | 35.2 |  | F |
| Intron CB-cox1-i | | 8554 | 8821 | 268 |  |  |  | 23.9 | 52.6/  23.5 |  |
| cox1 CDS2 | | 8822 | 9463 | 642 |  |  |  | 38.6 |  | F |
| Intron CB-cox1-ii | | 9464 | 9703 | 240 |  |  |  | 20.8 | 58.3/  20.8 |  |
| cox1 CDS3 | | 9704 | 10408 | 705 |  | UAA |  | 35.9 |  | F |
| Intron-encoded protein-like ORF | | 10463 | 11776 | 1314 | AUG | UAA |  | 29.2 |  | F |
| Group II reverse transcriptase | | <10745 | >11281 | 537 | ? | ? |  | 29.4 |  | F |
| Group II intron maturase | | <11384 | >11737 | 354 | ? | ? |  | 29.4 |  | F |
| trnI | | 11873 | 11940 | 68 |  |  | ATC | 26.5 |  | R |
| trnT | | 12004 | 12069 | 66 |  |  | ACA | 27.3 |  | R |
| trnS2 | | 12147 | 12203 | 57 |  |  | TCA | 29.1 |  | F |
| nad3 | | 12481 | 12831 | 351 | AUA |  |  | 29.9 |  | F |
| trnM | | 13029 | 13092 | 64 |  |  | ATG | 35.9 |  | F |
| rrnS | | 13104 | 13951 | 848 |  |  |  | 34.2 |  | F |
| cox2 CDS1 | | 13955 | 14080 | 126 | AUG |  |  | 36.5 |  | F |
| Intron CB-cox2-i | | 14081 | 14350 | 270 |  |  |  | 25.6 | 48.1/  26.3 |  |
| cox2 CDS2 | | 14351 | 14758 | 408 |  |  |  | 37.3 |  | F |
| Intron CB-cox2-ii | | 14759 | 14979 | 221 |  |  |  | 20.8 | 56.6/  22.6 |  |
| cox2 CDS3 | | 14980 | 15114 | 135 |  | UAA |  | 32.6 |  | F |
| trnY | | 15116 | 15164 | 49 |  |  | TAC | 36.7 |  | F |
| trnS1 | | 15214 | 15270 | 57 |  |  | AGA | 26.3 |  | F |
| trnQ | | 15291 | 15356 | 66 |  |  | CAA | 30.3 |  | F |
| trnG | | 15362 | 15428 | 67 |  |  | GGA | 40.3 |  | F |
| rrnL | | 15429 | 16686 | 1258 |  |  |  | 29.2 |  | F |
| trnF | | 16687 | 16752 | 66 |  |  | TTC | 34.8 |  | F |
| trnL1 | | 16762 | 16821 | 60 |  |  | CTA | 16.7 |  | F |
| trnK | | 16827 | 16893 | 67 |  |  | AAA | 34.3 |  | F |
| cox3 CDS1 | | 16915 | 17226 | 312 | AUG |  |  | 33.3 |  | F |
| Intron CB-cox3-i | | 17227 | 17472 | 246 |  |  |  | 22.4 | 53.3/  24.4 |  |
| cox3 CDS2 | | 17473 | 17808 | 336 |  |  |  | 40.5 |  | F |
| Intron CB-cox3-ii | | 17809 | 18076 | 268 |  |  |  | 30.2 | 52.2/  17,5 |  |
| cox3 CDS3 | | 18077 | 18205 | 129 |  | UAG |  | 39.5 |  | F |
| trnL2 | | 18495 | 18555 | 61 |  |  | TTA | 27.9 |  | F |
| trnP | | 18568 | 18638 | 71 |  |  | CCA | 26.8 |  | F |
| atp8 | | 18652 | 18765 | 114 | AUG | UAA |  | 28.9 |  | F |
| cytb CDS1 | | 18813 | 19079 | 267 | AUA |  |  | 34.8 |  | F |
| Intron CB-cytb-i | | 19080 | 19329 | 250 |  |  |  | 25.2 | 55.2/  19.6 |  |
| cytb CDS2 | | 19330 | 19701 | 372 |  |  |  | 37.1 |  | F |
| Intron CB-cytb-ii | | 19702 | 19968 | 267 |  |  |  | 27.3 | 51.3/  21.3 |  |
| cytb CDS3 | | 19969 | 20334 | 366 |  |  |  | 32.8 |  | F |
| Intron CB-cytb-iii | | 20335 | 20588 | 254 |  |  |  | 28.0 | 52.8/  19.3 |  |
| cytb CDS4 | | 20589 | 20705 | 117 |  | UAA |  | 30.8 |  | F |
| nad4L CDS1 | | 20772 | 20972 | 201 | AUG |  |  | 28.9 |  | F |
| Intron CB-nad4L | | 20973 | 21237 | 265 |  |  |  | 23.4 | 50.2/  26.4 |  |
| nad4L CDS2 | | 21238 | 21330 | 93 |  | UAA |  | 29.0 |  | F |
| nad4 CDS1 | | 21357 | 21599 | 243 | AUA |  |  | 27.2 |  | F |
| Intron CB-nad4-i | | 21600 | 21851 | 252 |  |  |  | 19.8 | 57.1/  23.0 |  |
| nad4 CDS2 | | 21852 | 21935 | 84 |  |  |  | 21.4 |  | F |
| Intron CB-nad4-ii | | 21936 | 22213 | 278 |  |  |  | 25.2 | 54.0/  20.9 |  |
| nad4 CDS3 | | 22214 | 23200 | 987 |  | UAA |  | 32.1 |  | F |

|  | ***Discoporella cookae* (AW3739; Contig A) 5,283 bp (linear); GenBank acc. MW592990** | | | | | | | | | |
| --- | --- | --- | --- | --- | --- | --- | --- | --- | --- | --- |
| **Gene** | | **From** | **To** | **Size (bp)** | **Start codon** | **Stop codon** | **tRNA**  **codon** | **GC (%)** | **A/T (%)** | **F/R** |
| Intron DC-nad6 | | <1 | 248 | 248 |  |  |  | 17.3 | 53.2/  29.4 |  |
| nad6 CDS^†^ | | 249 | 512 | 264 | ? | UAA |  | 20.8 |  | F |
| nad1 CDS1 | | 1655 | 1912 | 258 | AUU |  |  | 27.1 |  | F |
| Intron DC-nad1-i | | 1913 | 2179 | 267 |  |  |  | 16.9 | 50.9/  32.2 |  |
| nad1 CDS2 | | 2180 | 2311 | 132 |  |  |  | 36.4 |  | F |
| Intron DC-nad1-ii | | 2312 | 2577 | 266 |  |  |  | 20.7 | 50.8/  28.6 |  |
| nad1 CDS3 | | 2578 | 3033 | 456 |  | UAA |  | 31.4 |  | F |
| trnN | | 3286 | 3356 | 71 |  |  | AAC | 26.8 |  | F |
| trnM | | 3590 | 3652 | 63 |  |  | ATG | 41.3 |  | F |
| rrnS | | 3653 | 4519 | 867 |  |  |  | 28.3 |  | F |
| trnF | | 4660 | 4724 | 65 |  |  | TTC | 26.2 |  | F |
|  | ***Discoporella cookae* (AW3739; Contig B) 15,602 bp (partial); GenBank acc. MW592989** | | | | | | | | | |
| **Gene** | | **From** | **To** | **Size (bp)** | **Start codon** | **Stop codon** | **tRNA**  **codon** | **GC (%)** | **A/T (%)** | **F/R** |
| Intron DC-nad2 | | <1 | 397 | >397 |  |  |  | 24.4 | 47.6/  31.5 |  |
| nad2 CDS^†^ | | 398 | 637 | 240 | ? | UAA |  | 22.1 |  | F |
| nad5 CDS1 | | 703 | 1083 | 381 | AUG |  |  | 24.9 |  | F |
| Intron DC-nad5-i | | 1084 | 1375 | 292 |  |  |  | 21.2 | 46.9/  31.8 |  |
| nad5 CDS2 | | 1376 | 1561 | 186 |  |  |  | 21.5 |  | F |
| Intron DC-nad5-ii | | 1562 | 1809 | 248 |  |  |  | 19.8 | 49.2/  31.0 |  |
| nad5 CDS3 | | 1810 | 1905 | 96 |  |  |  | 44.8 |  | F |
| Intron DC-nad5-iii | | 1906 | 2145 | 240 |  |  |  | 14.2 | 51.3/  34.6 |  |
| nad5 CDS4 | | 2146 | 3183 | 1038 |  | UAA |  | 27.2 |  | F |
| trnD | | 3255 | 3322 | 68 |  |  | GAC | 22.1 |  | F |
| cox1 CDS1 | | 3335 | 3541 | 207 | AUG |  |  | 30.0 |  | F |
| Intron DC-cox1-i | | 3542 | 3818 | 277 |  |  |  | 19.1 | 49.5/  31.4 |  |
| cox1 CDS2 | | 3819 | 4460 | 642 |  |  |  | 35.8 |  | F |
| Intron DC-cox1-ii | | 4461 | 4705 | 245 |  |  |  | 19.6 | 53.1/  27.3 |  |
| cox1 CDS3 | | 4706 | 5419 | 714 |  | UAA |  | 31.7 |  | F |
| trnS2 | | 5462 | 5516 | 55 |  |  | TCA | 25.5 |  | F |
| cox2 CDS1 | | 5540 | 5665 | 126 | AUG |  |  | 25.4 |  | F |
| Intron DC-cox2-i | | 5666 | 5927 | 262 |  |  |  | 15.6 | 48.5/  35.9 |  |
| cox2 CDS2 | | 5928 | 6335 | 408 |  |  |  | 30.1 |  | F |
| Intron DC-cox2-ii | | 6336 | 6624 | 289 |  |  |  | 18.0 | 48.1/  33.9 |  |
| cox2 CDS3 | | 6625 | 6759 | 135 |  | UAA |  | 25.9 |  | F |
| trnQ | | 6828 | 6895 | 68 |  |  | CAA | 32.4 |  | F |
| trnG | | 6899 | 6966 | 68 |  |  | GGA | 26.5 |  | F |
| rrnL | | 6967 | 8214 | 1248 |  |  |  | 24.1 |  | F |
| trnI | | 8245 | 8311 | 67 |  |  | ATC | 20.9 |  | F |
| nad3 | | 8624 | 8953 | 330 | AUU |  |  | 23.6 |  | F |
| Intron-encoded protein-like ORF | | 8959 | 10293 | 1335 | AUA | UAA |  | 22.6 |  | F |
| Group II reverse transcriptase | | <9244 | >9789 | 546 | ? | ? |  | 24.5 |  | F |
| Group II intron maturase | | <9919 | >10275 | 357 | ? | ? |  | 23.0 |  | F |
| trnL1 | | 10338 | 10396 | 59 |  |  | CTA | 13.6 |  | F |
| trnK | | 10414 | 10484 | 71 |  |  | AAA | 38.0 |  | F |
| trnL2 | | 10486 | 10547 | 62 |  |  | TTA | 22.6 |  | F |
| trnY | | 10572 | 10629 | 58 |  |  | TAC | 31.0 |  | F |
| trnS1 | | 10632 | 10688 | 57 |  |  | AGA | 33.3 |  | F |
| cox3 CDS1 | | 10710 | 11021 | 312 | AUG |  |  | 33.7 |  | F |
| Intron DC-cox3-i | | 11022 | 11304 | 283 |  |  |  | 18.0 | 47.7/  34.3 |  |
| cox3 CDS2 | | 11305 | 11640 | 336 |  |  |  | 35.1 |  | F |
| Intron DC-cox3-ii | | 11641 | 11906 | 266 |  |  |  | 14.7 | 53.0/  32.3 |  |
| cox3 CDS3 | | 11907 | 12035 | 129 |  | UAA |  | 31.8 |  | F |
| trnP | | 12376 | 12438 | 63 |  |  | CCA | 25.4 |  | F |
| atp8 | | 12447 | 12560 | 114 | AUG | UAA |  | 21.9 |  | F |
| cytb CDS1 | | 12577 | 12843 | 267 | AUG |  |  | 30.7 |  | F |
| Intron DC-cytb-i | | 12844 | 13093 | 250 |  |  |  | 16.8 | 49.6/  33.6 |  |
| cytb CDS2 | | 13094 | 13465 | 372 |  |  |  | 30.6 |  | F |
| Intron DC-cytb-ii | | 13466 | 13747 | 282 |  |  |  | 19.1 | 48.2/  32.6 |  |
| cytb CDS3 | | 13748 | 14113 | 366 |  |  |  | 29.2 |  | F |
| Intron DC-cytb-iii | | 14114 | 14379 | 266 |  |  |  | 16.2 | 51.5/  32.3 |  |
| cytb CDS4 | | 14385 | 14477 | 93 |  | UAA |  | 24.7 |  | F |
| nad4L CDS1 | | 14557 | 14757 | 201 | AUG |  |  | 23.9 |  | F |
| Intron DC-nad4L | | 14758 | 15016 | 259 |  |  |  | 19.7 | 47.1/  33.2 |  |
| nad4L CDS2 | | 15017 | 15109 | 93 |  | UAA |  | 24.7 |  | F |

? precise CDS start/end unknown

* Abbreviated stop codon; UAA stop codon is completed by the addition of 3' A residues to the mRNA

^§‑^Putative initiation codons GUU which encode valine

< incomplete at 5’ end; > incomplete at 3’ end

† Unknown CDS number because gene is incomplete at the 5’ end

CDS = Coding sequence

F = Forward direction

R = Reverse direction
