# Supplementary material for: Unprecedented frequency of mitochondrial introns in colonial bilaterians
Source: Sci Rep. 2022 Jun 28;12:10889. doi: 10.1038/s41598-022-14477-3 (PMC9240083; doi:10.1038/s41598-022-14477-3)
Supplement: Supplementary file 12 — Supplementary Table S2. [file 41598_2022_14477_MOESM12_ESM.docx]

**Supplementary Table S2**. List of introns found in the mitogenomes of *Exechonella vieirai*, *Parantropora penelope*, *Cupuladria biporosa*, and *Discoporella* *cookae*. Capital prefixes of intron names represent genus/species initials. Whenever multiple introns were found per gene, introns are labelled with suffixes i-iii. Intron-encoded proteins (IEPs) with reverse transcriptase (RVT) and intron maturase (IM) domains are indicated. *Intron putatively interrupted by short nad5 ORF. **5’ end missing. ^‡^Intron verified by PCR. Standard start (GUGYG) and stop ([Y]AY) motifs are indicated in bold.

| **Taxon** | **Intron name** | **Intron type** | **Size (bp)** | **Start/end motifs** |
| --- | --- | --- | --- | --- |
| *Exechonella vieirai* | EV-cox1^‡^  IEP  RVT domain  IM domain | Group II | 2082  1539  705  342 | **GUGCG/UAC** |
|  | EV-cytb-i | Putative Group II | 581 | **GUGCG/CAC** |
|  | EV-cytb-ii^‡^ | Putative Group II | 527 | **GUGCG/CAC** |
|  | EV-cytb-iii | Putative Group II | 582 | **GUGCG**/**CAC** |
|  | EV-nad3^‡^ | Putative Group II | 824 | **GUGCG/UAC** |
|  | EV-H-nad5 | Putative Group II* | 1538 | **GUGCG/**CC |
|  | EV-nad5^‡^ |  | 266 | AGCCU**/CAC** |
|  | EV-atp6 | Putative Group II | 606 | **GUGCG/CAC** |
| *Parantropora penelope* | PP-cox1-i^‡^  IEP  RVT domain  IM domain | Group II | 2210  1569  429  288 | **GUGCG/UAC** |
|  | PP-cox1-ii^‡^ | Putative Group II | 527 | GUAUG/**UAC** |
|  | PP-cox2^‡^ | Putative Group II | 632 | **GUGCG/UAC** |
|  | PP-nad5 | Putative Group II | 632 | **GUGCG/UAC** |
|  | PP-cytb^‡^ | Putative Group II | 624 | **GUGCG/UAC** |
| *Cupuladria biporosa* | CB-cox1-i^‡^ |  | 268 | AACCU/**AC** |
|  | CB-cox1-ii^‡^ |  | 240 | AAACA/**AU** |
|  | IEP-like ORF  RVT domain  IM domain | N/A | 1314  537  354 | N/A |
|  | CB-cox2-i |  | 270 | AUUAA/UU |
|  | CB-cox2-ii^‡^ |  | 221 | UAGAA/CA |
|  | CB-cox3-i^‡^ |  | 246 | UAAAU/**AC** |
|  | CB-cox3-ii^‡^ |  | 268 | AACUA/**AU** |
|  | CB-cytb-i^‡^ |  | 250 | AAAAU/**AC** |
|  | CB-cytb-ii^‡^ |  | 267 | AAACC/**AC** |
|  | CB-cytb-iii |  | 254 | AAUCU/**AC** |
|  | CB-nad1-i^‡^ |  | 266 | AACCU/AA |
|  | CB-nad1-ii^‡^ |  | 262 | AAUAA/**AU** |
|  | CB-nad2^‡^ |  | 260 | AAAAC/AA |
|  | CB-nad4-i |  | 252 | AAAAC/**AU** |
|  | CB-nad4-ii |  | 278 | AAAAA/**AU** |
|  | CB-nad4L^‡^ |  | 265 | UUAAA/UA |
|  | CB-nad5-i^‡^ |  | 254 | UUUAA/AA |
|  | CB-nad5-ii^‡^ |  | 256 | CAACC/UA |
|  | CB-nad6^‡^ |  | 519 | GCUUA/**AU** |
| *Discoporella cookae* | D-cox1-i |  | 277 | AGAUG/AA |
|  | D-cox1-ii |  | 245 | AGGUG/AA |
|  | D-cox2-i |  | 262 | AUUAA/UU |
|  | D-cox2-ii |  | 289 | UGUAG/UA |
|  | D-cox3-i |  | 283 | AGGUG/**AC** |
|  | D-cox3-ii |  | 266 | AGACU/**AC** |
|  | D-cytb-i |  | 250 | AGGUG/AA |
|  | D-cytb-ii |  | 282 | AGGCG/**AU** |
|  | D-cytb-iii |  | 266 | UAGUG/**AU** |
|  | D-nad1-i |  | 267 | AGGUG/GU |
|  | D-nad1-ii |  | 266 | AAUAA/GU |
|  | D-nad2** |  | 397 | ?/**AU** |
|  | IEP-like ORF  RVT domain  IM domain | N/A | 1335  546  357 | N/A |
|  | D-nad4L |  | 259 | GUAUG/**AC** |
|  | D-nad5-i |  | 292 | UGACU/**AU** |
|  | D-nad5-ii |  | 248 | UAAUU/UA |
|  | D-nad5-iii |  | 240 | GAACA/AA |
|  | D-nad6** |  | 248 | ?/**AU** |
