# Supplementary material for: Unprecedented frequency of mitochondrial introns in colonial bilaterians
Source: Sci Rep. 2022 Jun 28;12:10889. doi: 10.1038/s41598-022-14477-3 (PMC9240083; doi:10.1038/s41598-022-14477-3)
Supplement: Supplementary file 14 — Supplementary Table S4. [file 41598_2022_14477_MOESM14_ESM.docx]

**Supplementary Table S4.** Specific primers designed and used in this study to confirm the presence of introns. PCR product sizes are given in ‘Size (bp)’. ‘T_ann_ (°C)’ are annealing temperatures used in PCR cycling, * denotes long-range PCRs. PCR numbers correspond to those shown in Figure 2 and Supplementary Figure S1.

| **Intron/primer names** | **Sequence (5’-3’) F – forward, R – reverse** | | **Size (bp)** | **T_ann_ (°C)** | **PCR number** |
| --- | --- | --- | --- | --- | --- |
| EV-cox1 |  |  |  |  |  |
| AW1260_cox1_a_F | F | CCCACATTATTGCTAACTACAGAAC | 2,236 | 51* | 1 |
| AW1260_cox1_b_R | R | TCTAAAGTAGGCTCGGGTGTC |  |  |  |
| EV-nad3 |  |  |  |  |  |
| AW1260_nad3_a_F | F | CACGACACCAACTAAAACGAG | 1,038 | 54 | 2 |
| AW1260_nad3_b_R | R | GGCTAAAAAAATGGCAGATG |  |  |  |
| EV-cytb-ii |  |  |  |  |  |
| AW1260_cytb_a_F | F | TAACCCGATTCTACGCACTCC | 816 | 55 | 3 |
| AW1260_cytb_b_R | R | TTCTGGTTGAATGTGTGTTGG |  |  |  |
| EV-H-nad5/EV-nad5 |  |  |  |  |  |
| AW1260_H-nad5_F | F | AACTCTCGCACCTACCCGATACT | 607 | 58 | 4 |
| AW1260_nad5_R | R | AGTGGAGTCTAACATGATGGGGATAG |  |  |  |
| PP-cytb |  |  |  |  |  |
| AW2102_cob_a_F | F | ATAAGGAAACCTGGATAGTGG | 769 | 51 | 5 |
| AW2102_cob_b_R | R | CACCCAAATAAGGAACAGC |  |  |  |
| PP-cox1-i |  |  |  |  |  |
| AW2102_cox1_a_F | F | GGTACTTTGTATTTTTTGTTTGGC | 2,571 | 51* | 6 |
| AW2102_cox1_b_R | R | ACATTGGCAGAAAGAGGCG |  |  |  |
| PP-cox1-ii |  |  |  |  |  |
| AW2102_cox1_b_F | F | TTACATTTGGGGGGCTGAC | 988 | 55 | 7 |
| AW2102_cox1_c_R | R | AGTCTAAAGGTGTGCGGTCC |  |  |  |
| PP-cox2 |  |  |  |  |  |
| AW2102_cox2_a_F | F | AAATGTCAGGATGGTCTCAAG | 832 | 52 | 8 |
| AW2101_cox2_b_R | R | AAATAGTCCACACCACCTCC |  |  |  |
| CB-nad6 |  |  |  |  |  |
| AW817_nad6_a_F | F | ACACTAATAATCCTTTCTGTAACCG | 911 | 54 | 9 |
| AW817_nad6_b_R | R | TAATGCTAAGAATAAGGTGATTGC |  |  |  |
| CB-nad1-i |  |  |  |  |  |
| AW817_nad1_a_F | F | TAACACAACCATTAGCCGACG | 572 | 52 | 10 |
| AW817_nad1_b_R | R | GCTATTCTGATTTCGTATGAGATTG |  |  |  |
| CB-nad1-ii |  |  |  |  |  |
| AW817_nad1_b_F | F | ATACTCTCTATTAGGAGCAACCC | 457 | 51 | 11 |
| AW817_nad1_c_R | R | GGCGATTCTGGTTACTATTCATAC |  |  |  |
| CB-nad2 |  |  |  |  |  |
| AW817_nad2_a_F | F | GCTGTATTATCCTAACAACTCTCC | 490 | 51 | 12 |
| AW817_nad2_b_R | R | GGCATAGGTTGATTGATTGTG |  |  |  |
| CB-nad5-i |  |  |  |  |  |
| AW817_nad5_a_F | F | TTGGATGAGACGGATTAGGAG | 382 | 52 | 13 |
| AW817_nad5_b_R | R | TTCTTAGGATTAGGAAGGCG |  |  |  |
| CB-nad5-ii |  |  |  |  |  |
| AW817_nad5_b_F | F | TTCCTAATCCTAAGAAGAGCCTG | 434 | 54 | 14 |
| AW817_nad5_c_R | R | GCTGATACAGGGGTAGGTGC |  |  |  |
| CB-cox1-i |  |  |  |  |  |
| AW817_cox1_a_F | F | TTAGCCCAACCAGGAAACC | 442 | 55 | 15 |
| AW817_cox1_b_R | R | TGTTTAGTCGGGGGAATGC |  |  |  |
| CB-cox1-ii |  |  |  |  |  |
| AW817_cox1_b_F | F | ATCCTTCTTTGACCCCGC | 670 | 54 | 16 |
| AW817_cox1_c_R | R | AGTTTGCTAATACGATGCCG |  |  |  |
| CB-cox2-ii |  |  |  |  |  |
| AW817_cox2_b_F | F | TAGCACTAAAAGCAGACGCAG | 326 | 55 | 17 |
| AW817_cox2_c_R | R | TTTGCTCCGCAGATTTCAG |  |  |  |
| CB-cox3-i |  |  |  |  |  |
| AW817_cox3_a_F | F | GATTTCATACATCCCACACCG | 647 | 55 | 18 |
| AW817_cox3_b_R | R | ATCCTGTTGCTACAAAGAATGTG |  |  |  |
| CB-cox3-ii |  |  |  |  |  |
| AW817_cox3_b_F | F | TCCCACAAAGAGACTACCTGAAG | 547 | 54 | 19 |
| AW817_cox3_c_R | R | CTACATCAACAAAGTGTCAGTATCAG |  |  |  |
| CB-cytb-i |  |  |  |  |  |
| AW817_cytb_a_F | F | AACGATAAGATACGACCCAAAC | 539 | 54 | 20 |
| AW817_cytb_b_R | R | TTGTAATGACGGTTGCTGCTC |  |  |  |
| CB-cytb-ii |  |  |  |  |  |
| AW817_cytb_b_F | F | CATACCGTTCGTCATCATAGC | 414 | 54 | 21 |
| AW817_cytb_c_R | R | ACAGAATCCAATCAGGTCTTTTAC |  |  |  |
| CB-nad4L |  |  |  |  |  |
| AW817_nad4L_a_F | F | TAATCAGAGCATTCTTCACACG | 476 | 54 | 22 |
| AW817_nad4L_b_R | R | ATCGTTTCCTTTGAATCGTG |  |  |  |
